# Phylogeny of the Eurasian Wren *Nannus troglodytes* (Aves: Passeriformes: Troglodytidae) reveals deep and complex diversification patterns of Ibero-Maghrebian and Cyrenaican populations

**DOI:** 10.1371/journal.pone.0230151

**Published:** 2020-03-19

**Authors:** Frederik Albrecht, Jens Hering, Elmar Fuchs, Juan Carlos Illera, Flora Ihlow, Thomas J. Shannon, J. Martin Collinson, Michael Wink, Jochen Martens, Martin Päckert

**Affiliations:** 1 Museum of Zoology, Senckenberg Natural History Collections Dresden, Senckenberg|Leibniz Institution for Biodiversity and Earth System Research, Dresden, Saxony, Germany; 2 Verein Sächsischer Ornithologen e.V., Limbach-Oberfrohna, Saxony, Germany; 3 Verein Sächsischer Ornithologen e.V., Weimar, Thuringia, Germany; 4 Research Unit of Biodiversity (UO-CSIC-PA), Oviedo University, Asturias, Spain; 5 School of Medicine, Medical Sciences and Nutrition, University of Aberdeen, Aberdeen, Scotland, United Kingdom; 6 Institute of Pharmacy and Molecular Biotechnology, Heidelberg University, Heidelberg, Baden-Württemberg, Germany; 7 Institute of Organismic and Molecular Evolution, Johannes Gutenberg University Mainz, Mainz, Rhineland-Palatinate, Germany; Institute of Systematics and Evolution of Animals Polish Academy of Sciences, POLAND

## Abstract

The Mediterranean Basin represents a Global Biodiversity Hotspot where many organisms show high inter- and intraspecific differentiation. Extant phylogeographic patterns of terrestrial circum-Mediterranean faunas were mainly shaped through Pleistocene range shifts and range fragmentations due to retreat into different glacial refugia. Thus, several extant Mediterranean bird species have diversified by surviving glaciations in different hospitable refugia and subsequently expanded their distribution ranges during the Holocene. Such a scenario was also suggested for the Eurasian Wren (*Nannus troglodytes*) despite the lack of genetic data for most Mediterranean subspecies. Our phylogenetic multi-locus analysis comprised 18 out of 28 currently accepted subspecies of *N*. *troglodytes*, including all but one subspecies which are present in the Mediterranean Basin. The resulting phylogenetic reconstruction dated the onset of the entire Holarctic radiation of three *Nannus* species to the early Pleistocene. In the Eurasian Wren, two North African subspecies represented separate basal lineages from the Maghreb (*N*. *t*. *kabylorum*) and from the Libyan Cyrenaica (*N*. *t*. *juniperi*), being only distantly related to other Mediterranean populations. Although *N*. *troglodytes* appeared to be paraphyletic with respect to the Nearctic Winter Wren (*N*. *hiemalis*), respective nodes did not receive strong statistical support. In contrast, paraphyly of the Ibero-Maghrebian taxon *N*. *t*. *kabylorum* was strongly supported. Southern Iberian populations of *N*. *t*. *kabylorum* did not clade with Maghrebian populations of the same subspecies but formed a sister clade to a highly diverse European clade (including nominate *N*. *t*. *troglodytes* and eight further taxa). In accordance with a pattern also found in other birds, Eurasian populations were split into a western clade (Europe, Caucasus) and an eastern clade (Central Asia, Sino-Himalayas, East Asia). This complex phylogeographic pattern revealed cryptic diversification in *N*. *troglodytes*, especially in the Iberio-Maghrebian region.

## Introduction

In the Western Palearctic, the Mediterranean Basin represents a region of exceptional genetic and species diversity both of flora and fauna and it is therefore recognized as a Global Biodiversity Hotspot [1–5]. Due to the colonization of structurally very different peninsulas (Iberian, Apennine, Balkan, and Anatolian Peninsulas), island archipelagos (of the Mediterranean Sea and Macaronesia), and the North African coastline, many species occur in marginal or isolated distributional areas with regard to their European core distributions [3,6,7]. At a more narrow spatial scale, the Mediterranean Basin comprises multiple regional vegetational Biodiversity sub-Hotspots [8–10] which largely coincide with several climatically stable refugia, where many endemic species have survived during the Pliocene and Pleistocene [11–13].

Within the western Mediterranean Basin, the Ibero-Maghrebian Region (IMR) comprises the Iberian Peninsula and the Maghreb Region of Northwest Africa. The latter stretches from the northernmost part of the Western Sahara Territory over Morocco and northern Algeria to Tunisia [14,15]. The IMR has previously been described in different scientific contexts, such as seismotectonics [16–18] and biogeography [19–23]. It furthermore includes the three Biodiversity sub-Hotspots of the High and Middle Atlas, the Baetic–Rifan mountain complex, and Kabylia–Numidia–Kroumire and is neighboured by the Mediterranean Cyrenaica of northeast Libya [9,12]. Being part of the Mediterranean biome, the IMR is characterized by Mediterranean sclerophyllous forests, woodlands, and scrub, but also by continued Palearctic influence, both in floral and in faunal communities [3]. For examples, several Euro-Siberian tree and shrub species reach their southernmost range limits here in the form of Maghrebian exclaves (such as *Taxus baccata*, *Ilex aquifolium*, *Sorbus aria*, *Prunus avium*, *Populus tremula*, *Acer campestre* [6,24]). A commonly observed phylogeographical pattern in many vertebrate taxa of the IMR is a strong differentiation between Iberian and Maghrebian populations and in many cases, also a further differentiation of western and eastern Maghrebian populations (e.g. reviewed for amphibians and reptiles [15,25,26]). Further east in the Mediterranean Cyrenaica, small relict populations of bush- and forest-dwelling bird species are known to exist on the forested Jebel Akhdar massif. These are separated by a large desert area from their closest conspecifics in the Maghreb and therefore, some Cyrenaican populations represent distinct and relict genetic lineages (e.g. in African Blue Tits *Cyanistes teneriffae* [27–29] and in Common Chaffinches *Fringilla coelebs* [30]).

The Eurasian Wren *Nannus troglodytes* (until recently referred to as *Troglodytes troglodytes*; see explanation below) inhabits the IMR at the south-western periphery of its Palearctic-wide distributional range. Here, it prefers forest and shrubland habitats with dense undergrowth, often in the proximity of watercourses [31–34], such as forested stream valleys at higher elevations in North Africa [32]. It is the only Palearctic species of the otherwise Nearctic and Neotropic family of wrens (Passeriformes: Troglodytidae), which currently comprises up to 93 recognised species [31–33]. Conventionally, all Holarctic populations of the “Winter Wren” or “Northern Wren” had been united under the species-level taxon *Troglodytes troglodytes* [31,32,34], until Drovetski and colleagues [35] demonstrated that Palearctic and Nearctic populations are divided into separate mitochondrial DNA (mtDNA) lineages. As this genetic divergence was paralleled by differences in territorial songs [36], a species-level split of Palearctic from Nearctic “Winter Wren” populations was recommended [37]. Today, most authorities accordingly recognize the Eurasian Wren *T*. *troglodytes* as an exclusively Palearctic species [33,38,39]. For its remaining Nearctic relatives, a high level of genetic divergence between eastern and western populations is accompanied by slight but consistent bioacoustic differentiation [40,41] and due to a lack of vocal admixture in an area of sympatry, reproductive isolation has been assumed [42]. Therefore, the Nearctic “Winter Wren” populations were furthermore split into two species: the Pacific Wren *T*. *pacificus* of the Western Nearctic and the Winter Wren *T*. *hiemalis* of the Eastern Nearctic [33,37,38].

According to a recent molecular study [43], there is increasing evidence that the genus *Troglodytes* (*sensu* e.g. [31–33]) is not monophyletic. Therefore it was recommended to transfer *T*. *troglodytes*, *T*. *pacificus*, and *T*. *hiemalis* into the re-established genus *Nannus* Billberg, 1828 (as already suggested before by [44,45]), and to restrict the genus name *Troglodytes* Vieillot, 1809, to a monophyletic group of New World taxa (see [46], as referenced in [38]). Although these taxonomic recommendations have not yet been implemented into a major taxonomic compendium, we assume that this will be inevitably the case in the near future and we therefore follow the suggestion by Barker [43] and henceforth refer to the spp. *troglodytes*, *pacificus*, and *hiemalis* as members of the genus *Nannus*.

The Eurasian Wren is highly polytypic and currently populations of *N*. *troglodytes* are assigned to 28 (the preferred concept throughout this study [38,39,47]) or to 29 [33] subspecies, showing the highest subspecies diversity in Europe (North Atlantic islands and Mediterranean Basin) and in Eastern Asia (China and northwest Pacific islands from Taiwan to Kamchatka). In both regions, a differentiation into island-endemic subspecies subtly differing in size-proportions, plumage-barring, and coloration can be observed [31,32,48].

Based on an analysis of mitochondrial *NADH dehydrogenase 2* (*ND2*), the taxonomic diversity of *N*. *troglodytes* corresponds to four separate mitochondrial lineages, comprising six subspecies so far documented by Drovetski *et al*. [35]: A European lineage (sspp. *troglodytes* and *indigenus*), a Caucasian (ssp. *hyrcanus*), a Nepalese (ssp. *nipalensis*), and an East Asian lineage (sspp. *dauricus* and *fumigatus*). Further molecular genetic analyses of North Atlantic island populations (sspp. *islandicus*, *borealis*, *zetlandicus*, *fridariensis*, *hirtensis*, *hebridensis*) found only small genetic divergence of these subspecies from populations of ssp. *indigenus* from Great Britain and Ireland and from continental European nominate *troglodytes*, suggesting recent differentiation [49,50]. All North Atlantic island subspecies thus originated from Palearctic rather than from Nearctic founders and did not substantially diverge since the colonization event [49]. Wren populations of the northernmost ranges in Europe and East Asia are migratory and leave their breeding grounds in winter, whereas remaining populations are largely sedentary to partially migratory [31,32].

Although previous studies [35,49,50] shed light on the intraspecific differentiation of the Eurasian Wren, a broader geographic and taxonomic coverage is much required. Also the phylogenetic relationships of *N*. *troglodytes* taxa distributed in the IMR within the Mediterranean Biodiversity Hotspot remain unknown. Due to their peripheral and fragmented range with regard to the Palearctic core distribution they might, however, yield important information on the biogeographic history of this species.

In this study, we aim to scrutinize the genetic structure of *N*. *troglodytes* populations based on a denser taxon sampling at the subspecies level. Our focus was set on the phylogenetic relationships of unstudied populations from the forested margins of the Qinghai-Tibet Plateau (i.e. sspp. *talifuensis* and *idius*) and of Ibero-Maghrebian wren populations, represented by the isolated and poorly investigated subspecies *N*. *t*. *juniperi* of the Cyrenaica [51–54], by *N*. *t*. *kabylorum* of Northwest Africa, the Balearic Islands, and southern Iberia, by *N*. *t*. *koenigi* endemic to Corsica and Sardinia, and by the nominate form *N*. *t*. *troglodytes* of northern Iberia and the rest of the European mainland. As phylogeographic results based on mtDNA data alone do not necessarily need to be consistent with findings from nuclear DNA analyses [55], our phylogenetic analysis relied on two mitochondrial genes and three nuclear markers (two introns, one exon) for 18 out of the 28 subspecies, of which six subspecies have not been subject to genetic analysis to date.

## Materials and methods

### Sampling and laboratory procedures

**Fig 1:**
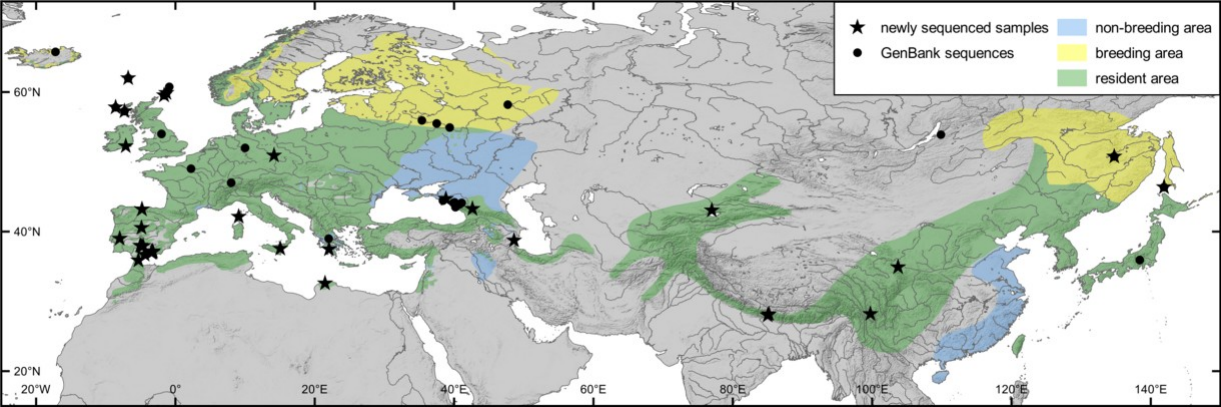
Distributional area of *Nannus troglodytes* in the Palearctic. Seasonal ranges indicated by colour (green: whole-year resident; yellow: breeding; blue: non-breeding; data from [56]), and genetic sampling localities for our phylogenetic analyses indicated by symbols (stars: newly sequenced samples for this study; circles: sequences retrieved from GenBank).

We analysed a total of 45 tissue and blood samples from 35 localities in the Palearctic (Fig 1). For a list of used samples and GenBank accession numbers of newly analysed sequences (MN919550-MN919644; MN927043-MN927084; MN931755-MN931781) see S1 Table. Genetic sample material used for this study was obtained from the collections of natural history museums (NMS, MNHN, SNSD, UWBM). All sampling procedures are in compliance with animal research ethical guidelines of respective institutes, as well as with national guidelines of respective countries. Therefore, the study has not been formally approved by an animal research ethics committee. Material was specifically collected for this study i) in the UK (feathers lost during routine ringing operations licenced by the British Trust for Ornithology, thus not falling under animal care regulation; c.f. [49]), ii) in Spain, where field work was conducted under permits of the Council of Government of the Principality of Asturias (2013/001891) and of the Regional Government of Andalusia (ENSN/BRL//MCF). The dataset was completed with sequences of further ingroup as well as outgroup taxa archived in GenBank.

Total genomic DNA was extracted from ethanol- or buffer-preserved tissue and blood samples using an innuPREP DNA Mini Kit (Analytik Jena AG) or an innuPREP Blood DNA Mini Kit (Analytik Jena AG) respectively, following the manufacturer’s protocol except for overnight incubation with proteinase K for cell lysis in both procedures. We also included DNA extracts from eight samples of North Atlantic island populations previously analysed by Shannon *et al*. [49].

For identification of mitochondrial lineages, we amplified and sequenced two mitochondrial genes: barcoding standard marker *cytochrome oxidase subunit I* (*COI*, 696 bp) [57] and *NADH dehydrogenase subunit 2* (*ND2*, 1033 bp). We sequenced *ND2* for 35 samples for comparison with sequence data sets available from previous studies (112 sequences; see below and S1 Table). To reconstruct a most taxon-complete multi-locus phylogeny of *Nannus* wrens, we sequenced three further nuclear markers for at least one sample of each subspecific taxon (or mitochondrial lineage per taxon): *beta-fibrinogen gene*, *intron 5* (*Fib5*, 570 bp), *myoglobin gene*, *intron 2* (*Myo2*, 736 bp), and one partial exon of the *recombination activating protein 1 gene* (*RAG1*, 763 bp) (see S2 Table for used primers and PCR protocols). To hydrolyse surplus primers and nucleotides, PCR products were purified using ExoSAP-IT (Thermo Fisher Scientific, Waltham, MA, USA) according to the manufacturer’s instructions (adding 0.2 ml ExoSAP-IT solution in 4 ml H_2_O to each sample; thermocycler incubation with 37°C for 30 min, 94°C for 15 min).

PCR products were prepared for sequencing with BigDyeTM 3.1 Dye Terminator Cycle Sequencing Kits (Applied Biosystems, now at Thermo Fisher Scientific, Waltham, MA, USA), according to the manufacturers’ instructions. Cycle sequencing products were purified by using Sephadex (GE Healthcare, Munich, Germany), and sequenced in both reading directions on an ABI 3130xl DNA sequencer (Thermo Fisher Scientific, Waltham, MA, USA).

For each of the five markers, a sequence alignment was compiled in MEGA6 [58]: Forward and reverse DNA sequences for each individual sample were manually inspected and edited in a cross-check with the respective chromatogram signals (using Chromas v.2.6.5 (Technelysium Pty Ltd, Brisbane, Australia)). Sequences of both reading directions were then combined to a single consensus sequence per marker per sample. Further sequences of *Nannus* and *Troglodytes* taxa were imported from GenBank into the respective alignments to increase sample size and taxon coverage. For *ND2* and *COI* haplotype network reconstruction (see below), we included published sequences from GenBank into our datasets (see S1 Table). Furthermore, we included sequence data of at least one representative of all other families of Certhioidea (Sittidae, Certhiidae, Polioptidae) to the Troglodytidae data set (for full account of included sequences for phylogenetic analyses, see S1 Table). For hierarchical outgroup rooting we used sequence data of the Bohemian Waxwing (*Bombycilla garrulus*) and the Goldcrest (*Regulus regulus*).

### Phylogenetic analyses

#### Single-locus analyses

For inference of phylogeographic structure, we reconstructed haplotype networks for all markers using PopART v.1.7 [59]. To determine allele sequences of nuclear markers in heterozygote individuals, we applied the PHASE algorithm as implemented in DNA Sequence Polymorphism v.6 (DnaSP 6 [60]), with MCMC options at their default values (number of iterations: 100; thinning interval: 1; burn-in iterations: 100). Allelic haplotype networks were then created as TCS networks [61,62], using PopART v.1.7 [59] with gaps being treated as a 5^th^ state. For nuclear markers, we included sequences of *Troglodytes aedon* from GenBank as outgroups, as well as sequences of *Cistothorus palustris* and *C*. *platensis* published by Robbins and Nyári [63] for the marker *Fib5*.

#### Multi-locus analysis and divergence time estimates

We used Partitionfinder v.1.1.1 [64] to determine the best-fitting partitioning scheme and models of sequence evolution. The search for the best strategy was performed using the ‘beast’ model-set and heuristic search. The best fitting strategy according to the AICc criterion was a 9-partition scheme with both mitochondrial genes being split by codon positions and the three nuclear markers as separate partitions each (resulting partitioning scheme and corresponding substitution models: *COI*: 1^st^ position: GTR+Γ+I, 2^nd^ position: HKY+I, 3^rd^ position: GTR+I; *ND2*: 1^st^ position: TrN+Γ+I, 2^nd^ position: GTR+Γ+I, 3^rd^ position: GTR+Γ; *Fib5*: GTR+Γ+I; *Myo2*: HKY+Γ; *RAG1*: TrN+I).

A multi-locus tree was reconstructed using Bayesian inference of phylogeny in BEAST v.1.8.1 [65]. According to the best-fitting scheme, nine partitions were assigned to the five markers and the best-fitting models applied to each partition. All tree models were linked to one tree model and a ‘Speciation: Birth-Death Incomplete Sampling’ (BD model) tree prior was applied [66]. We optimized ESS values in exploratory runs with BEAST using different chain lengths and priors. It turned out that robustness of the analysis was most strongly influenced by prior choice of the six rate parameters of the GTR model: We obtained poor ESS values across all parameters when a gamma distribution (default setting) was applied to rate priors; however, ESS values were greatly improved when a uniform prior distribution was applied. Tree-model choice did not affect tree topology at all in exploratory runs under the Yule model (compare [67,68]). Divergence time estimates were slightly older under the Yule model than under the BD model; however, 95% HPD intervals for the time of the most recent common ancestor (tmrca) largely overlapped among runs under different tree models (compare [69]).

MCMC chains ran for 50,000,000 generations in two parallel runs, each with one cold and three heated chains (heating parameter λ = 0.1). Trees were sampled every 5,000^th^ generation. The first 3,000 samples were discarded as burn-in and model parameters and posterior probabilities were estimated from the remaining samples. Remaining trees were summarized in a 50% majority rule consensus tree. Effective sample sizes for priors used in the Bayesian inference of phylogeny were controlled in Tracer v.1.7.1 [70].The BEAST analysis was run three times using different seeds and convergence of BEAST runs was assessed after combining the three log files using LogCombiner v.1.7.1 into a single log file. The combined log file was inspected in Tracer v.1.7.1 and ESS values were checked for all parameters.

In addition, we reconstructed a multi-locus phylogeny using Maximum Likelihood (ML) with RAxML v.7.2.6 [71], using the GUI python application v.0.93 [72]. Node support in a ML framework was obtained by 1,000 bootstrap replicates with RAxML (thorough bootstrap option, 100 replicates). In two separate runs, we partitioned the concatenated matrix (5 partitions by gene; 9 partitions by gene and codon, see above) and applied the GTR+Γ+I model across partitions.

**Fig 2:**
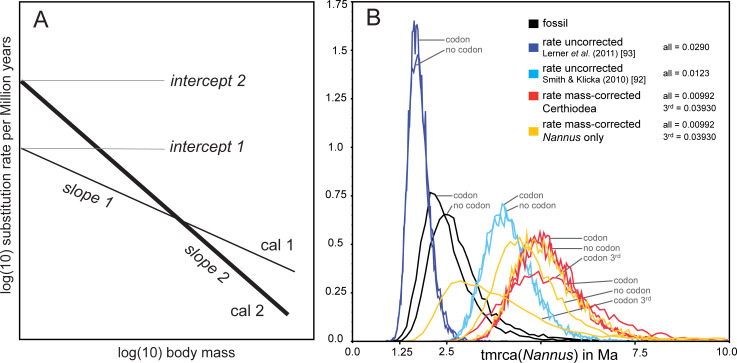
Inference of divergence time estimates from mitochondrial substitution rates. A: linear correlation between body mass and mitochondrial substitution rates in birds according to two calibration sets (cal1, cal2) by Nabholz *et al*. [99]; mass-corrected rates can be inferred from two parameters of the regression lines: slope and intercept; B: divergence time estimates for the time of the most recent common ancestor (tmrca) of *Nannus* according to 12 independent runs with BEAST; marginal density plots inferred from log output files with Tracer v.1.7.1; divergence time estimates were inferred from i) fossil calibration (by gene: *ND2* treated as a single partition; by codon: *ND2* partitioned by codon position), ii) two uncorrected rates for *ND2* (from [92] and [93], each partitioned by gene or by codon; see above), iii) a mass-corrected rate for the entire *ND2* gene of *Nannus* (partitioned by gene or by codon; see above), and iv) a mass corrected rate for the 3^rd^ codon position of *ND2* in *Nannu*s (partitioned by codon; see above); rates in substitutions per site per lineage per Ma indicated at upper left.

To estimate phylogenetic divergence times, we applied two methodological approaches in the form of i) molecular clock calibrations based on empirical substitution rates, and ii) a fossil time calibration. The latter was based on two fossil taxa of the superfamily Certhioidea (defined by [73]). Two fossils have been postulated as common ancestors of all Certhioidea, *Certhiops rummeli* Manegold, 2008 [74], from the early Miocene of Germany (MN3 ≙ 20.5–18.0 Ma), and the recently described taxon *Kischinskinia scandens* Volkova & Zelenkov, 2018 [75], from the early Miocene of eastern Siberia (MN5 ≙ 16.0–13.8 Ma [76]). The fossil age of the older fossil (*Certhiops*) was applied as estimate for the tmrca to the node uniting all Certhioidea. We performed our calibration according to the standard outlined by Benton *et al*. [77], who recommended the use of a soft maximum and minimum constraint that correspond to the oldest certain and the oldest possible date of origin of a clade. According to this approach, Claramunt and Cracraft [78] generated clade age priors for time calibration. Therefore we used their tmrca priors for Certhioidea for our fossil calibration: zero offset = 18.0; Log(mean) = 2.0; Log(stdev) = 1.2 (thus the known fossil age 20.5–18.0 Ma covered the time interval from the zero offset to the maximum of the lognormal prior distribution (c.f. [77]: Fig 2; [78]: Fig 1). Because monophyly of Certhioidea was strongly supported in previous analyses [79,80], the calibrated node was forced to be monophyletic. The geologic time scale applied in our analysis followed Walker *et al*. [81]. The trees obtained from the BEAST analysis were summarised to one consensus tree applying TreeAnnotator v1.8.1, using a burn-in set to a number of 3,000 trees and mean node heights. The final consensus trees were visualised in FigTree v.1.4.3 [82] with posterior probabilities (PP) and ML bootstrap as node support values.

For comparison, we inferred divergence time estimates based on empirical substitution rates for *ND2* from multiple independent runs with BEAST under a relaxed lognormal clock model. Some previous studies have considered the empirical substitution rate for the avian cytochrome-*b* (0.0105 substitutions per site per lineage per Ma [83]) as a ‘universal’ value for mtDNA in general [84]. However, this approach is problematic as substitution rates vary greatly among mitochondrial genes (birds: [85–87]; other vertebrates: [88,89]) and the substitution rate of a selected mtDNA marker may vary drastically among different bird orders with passerine mtDNA evolving fastest [90–92]. Divergence time estimates for *Nannus* wrens were previously inferred using an empirical rate based on a molecular clock calibration for *ND2* of Galapagos mockingbirds of 0.0276 substitutions per site per lineage per million years [35]. However, empirical rate estimates for *ND2* cover a rather broad range from 0.0123 to 0.029 subst/site/lin/Ma [93–96]. We therefore performed independent runs with BEAST based on the latter empirical maximum and minimum estimates. Moreover, there is firm evidence that in birds mitochondrial substitution rates are correlated with body size, body mass, and generation time [97–99], so that systematic biases could arise if rates are not corrected for different species (but see [92] for a critical reappraisal). Because the Eurasian Wren is one of the smallest Palearctic birds, we expect a considerable deviation from empirical mean mtDNA substitution rates for this species. We therefore calculated body mass-corrected substitution rates for *ND2* of wrens according to the parameters inferred from two different calibration sets (2 and 4) by Nabholz *et al*. [99] (Fig 2). We assumed a mean body mass of *Nannus* wrens of 9 g [48]. We used mass-corrected rates of the entire *ND2* gene and for the 3^rd^ codon position of *ND2* for further independent inference of divergence time estimates with BEAST. For reliable comparison with divergence time estimates based on the fossil calibration, we performed molecular clock calibrations using the entire Certhioidea data set of the *ND2* data set including all further outgroups. However, Nabholz *et al*. [99] recommended limiting data sets for molecular clock calibration using mass-corrected rates to closely related species of comparable body mass values. Because many other Certhioidea species are considerably larger than wrens (e.g. several nuthatches), we performed alternative runs with BEAST using only the data set of Troglodytidae (with sequences of *Polioptila caerulea* for hierarchical outgroup rooting).

According to molecular clock calibrations based on calibration set 2 [99] time estimates for the most recent common ancestor (tmrca) of Certhioidea were nearly as old as the most ancient known stem fossil of all Passeriformes (tmrca of Certhioidea: 26–29 Ma; fossil age: 30–34 Ma, [100,101]). We do not consider this a reasonable result and one reliable explanation is that time calibrations relying on calibration points only for outgroup taxa or near the root of a phylogenetic tree tend to overestimate divergence times particularly for crown groups of a phylogeny [102,103]. We consider calibration set 4 more appropriate for passerine birds, as it included one further calibration point within Passeriformes that was not used in calibration set 2. Therefore, in the following we do not consider mass-corrected rates inferred from calibration set 2 and show only results inferred from calibration set 4 by Nabholz *et al*. [99].

### Species Distribution Modelling (SDM)

To characterize the current distributional range of *Nannus troglodytes* based on its climatic niche and to draw conclusions on possible refugia of this species during the Last Glacial Maximum (LGM, ~ 22,000 years ago [104]), we performed species distribution models (SDMs) based on locality data covering the entire distributional range of this species and on a set of environmental predictor variables. Locality data was obtained in the form of geo-referenced collection data of preserved specimens from the online databases GBIF [105] and VertNet [106] (manually controlled for obvious mistakes, e.g. offshore occurrence, and corrected if possible), together with further occurrence data from the SNSD bird collection and type localities inferred from the literature [107]. Occurrence records based on geo-referenced tissue samples from earlier studies [35] and from the dataset compiled for this study (see Fig 1) were also included in the locality dataset. The dataset was spatially rarefied to a Euclidian distance of 5 km using the SDMtoolbox v1.1c [108] in ArcGIS v10.3 to prevent false inflation of model performance. Occurrences from assumed non-breeding wintering areas of partly migratory Eurasian Wrens in the Ponto–Caspian steppe (eastern Ukraine / southwest Russia) and in southeast China (Fig 1) were not included in the dataset.

As a result 583 unique records were retained for modelling. The following uncorrelated (correlation coefficients: R^2^ < 0.75) bioclimatic variables with a spatial resolution of 2.5 arc-minutes (~4 km at the equator) describing annual trends, seasonality, and limiting factors related to temperature and precipitation, were obtained from http://worldclim.org [104]: mean diurnal temperature range (bio 2), isothermality (bio 3), temperature seasonality (bio 4), maximum temperature of the warmest month (bio 5), mean temperature of the wettest quarter (bio 8), annual precipitation (bio 12), precipitation seasonality (bio 15), and precipitation of the driest quarter (bio 17). To assess the influence of past climate and sea level fluctuations on the distribution of *N*. *troglodytes* corresponding predictor variables for three projections for the LGM, derived from global circulation models through the Paleoclimate Modelling Intercomparison Project Phase II [109], were obtained [104]. These include the Community Climate System Model (CCSM3) [110], the Max-Planck-Institute Earth System Model P (MPI-ESM-P), and the Model for Interdisciplinary Research on Climate (MIROC) [111].

A circular buffer of 400 km surrounding each locality was used as background for model training whereas a rectangular study extent was selected as projection area. Therefore, the model was trained across the entire breeding range of *N*. *troglodytes* but projections were restricted to a smaller extent focussing on the Western Palearctic (Europe + North Africa). By doing so, we accounted for a sampling bias between relatively many records in the Western Palearctic and relatively few records in the Eastern Palearctic. Models were computed using the machine learning algorithm Maxent v3.4.1 [112–114]. Maxent is one of the most efficient presence-only data modelling tools. The feature classes linear, quadratic, and hinge were selected. A bootstrapping method with 100 replicates randomly splitting the data set into a training (80%) and a testing subset (20%) was applied. Subsequently, the model was projected onto the three LGM projections. The area under the curve (AUC), a threshold-independent measure of model performance, was used for model evaluation [115]. An AUC score of 1 refers to a perfect fit of the data while a score of 0.5 is no better than random [112,116]. The average projection across all replicate runs was used for further processing, wherein the minimum training presence logistic threshold was applied as presence-absence threshold.

## Results

### Single-locus reconstructions

**Fig 3:**
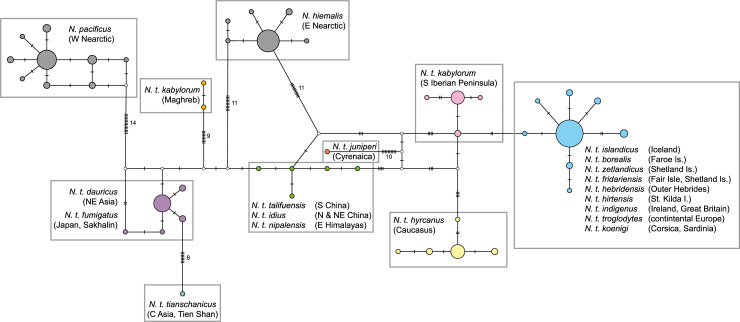
TCS haplotype network drawn for the mitochondrial *ND2* of the *Nannus pacificus/hiemalis/troglodytes* species complex including 18 subspecies of *N*. *troglodytes*. Haplotype circles scaled to sample size (n) of each represented haplotype (total sampling: n = 174); substitution distances not to scale.

The haplotype network based on mitochondrial *ND2* sequences (Fig 3; n = 174, sequence length: 723 bp) included the highest number of polymorphic sites among all markers used for this study and showed a differentiation of sequences into 46 haplotypes. Within this network, the Nearctic species *Nannus pacificus* and *N*. *hiemalis* were found in two independent genetic clusters at different positions; both species are remarkably differentiated from any neighbouring Palearctic clusters. For the Palearctic *N*. *troglodytes*, eight haplotype clusters could be identified as listed below:

1. **Widespread Western Palearctic.** This haplotype cluster comprised most of the Western Palearctic samples and included the nominate subspecies *N*. *t*. *troglodytes* together with seven insular subspecies from the North Atlantic Ocean (sspp. *islandicus*, *borealis*, *zetlandicus*, *fridariensis*, *hebridensis*, *hirtensis*, *indigenus*) as well as the Mediterranean subspecies *koenigi* (represented by two samples from Corsica sharing the same haplotype). The most common central haplotype (out of eight) of this cluster was found in 36 individuals from six subspecies. Three samples of nominate *N*. *t*. *troglodytes* from northern Spain (Picos de Europa) were also included in this cluster.
2. **Southern Iberia.** Samples of ssp. *kabylorum* from central and southern parts of the Iberian Peninsula (from Sotalbo, Province of Ávila, and from the Sierra Nevada, respectively) belonged to a separate mitochondrial cluster. This southern Iberian cluster comprised four haplotypes that differed from the central haplotype of the Western Palearctic cluster by at least three substitutions.
3. **Maghreb.** Strikingly, *N*. *t*. *kabylorum* populations from central and southern Iberia and those from Northwest Africa were not closely related; the two haplotype clusters differed by at least 20 substitutions.
4. **Cyrenaica.** The single haplotype of the isolated Cyrenaican population of *N*. *t*. *juniperi* did not fall into any other Mediterranean cluster but was placed in a separate cluster.
5. **Caucasus.** Further east on the Eurasian continent, individuals of *N*. *t*. *hyrcanus* from the Caucasus region formed a fifth cluster (of five haplotypes) that differed by at least seven substitutions from the Western Palearctic nominate cluster.
6. **Central Asia.** One haplotype of the Central Asian *N*. *t*. *tianschanicus* represented a sixth mitochondrial cluster that differed by at least eight substitutions from its nearest neighbouring cluster below.
7. **Northeast Asia.** Five haplotypes of the Northeast Asian and Japanese sspp. *dauricus* and *fumigatus* were assembled in this cluster. They differed by eight substitutions from the Central Asian haplotype of *N*. *t*. *tianschanicus* and by at least four substitutions from haplotypes of the Sino-Himalayan cluster.
8. **Sino-Himalayan.** This Asian cluster of the Sino-Himalayan region contained the haplotypes of Chinese sspp. *idius* (two haplotypes) and *talifuensis* (one haplotype), together with two Nepalese haplotypes of ssp. *nipalensis* at a distance of at least six substitutions.

A haplotype network based on the barcoding marker *COI* included 38 haplotypes of 18 subspecies of *N*. *troglodytes* which were allocated to the same clusters that were also identified by the *ND2* network (S1 Fig; n = 81, sequence length: 551 bp). Furthermore, the barcoding network included two samples which each represent an additional subspecies not included in the *ND2* haplotype network or in the multi-locus phylogeny: *N*. *t*. *cypriotes*, which was attributed to the cluster of *N*.*t*. *hyrcanus* from the Caucasus, and *N*. *t*. *mosukei* from the Izu Islands of Japan, sharing the most common haplotype within *N*. *t*. *fumigatus* (S1 Fig).

**Fig 4:**
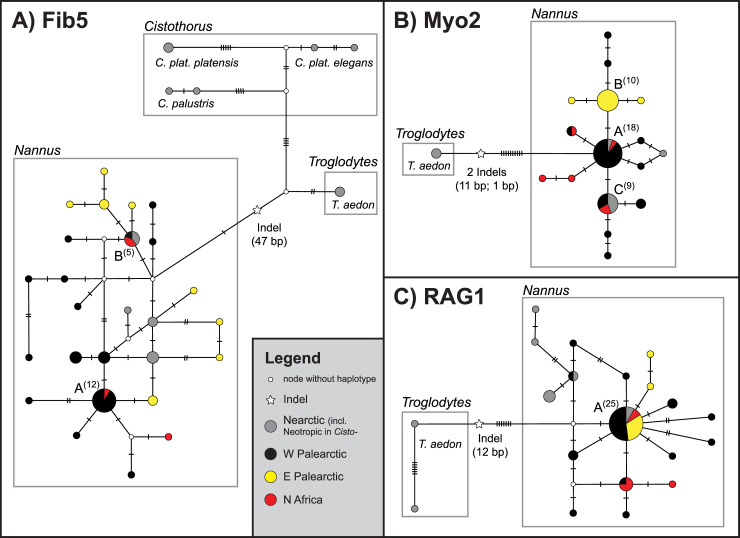
Allelic haplotype networks for three nuclear markers of *Nannus* spp. samples with *troglodytes aedon* as outgroup. A: *Fib5*, including *Cistothorus palustris* and *C*. *platensis* as outgroups (n = 31); B: *Myo2* (n = 27); C: *RAG1* (n = 26). Circles scaled to sample size (stated in parentheses for named allelic haplotypes); substitution distances not to scale.

Allelic haplotype networks for the three nuclear markers are shown in Fig 4. All three markers showed a pronounced differentiation of *Troglodytes aedon* alleles from alleles of the *Nannus* species complex, including indels of several base pairs (bp) in all three cases. For *Fib5*, *Cistothorus* and *T*. *aedon* were separated by a shared indel of 47 bp from *Nannus* (Fig 4A). None of the nuclear markers showed a clear phylogeographic structure within *Nannus* and many alleles were shared among regions (e.g. *Fib5* allele B, *Myo2* alleles A and C, *RAG1* allele A; Fig 4). Only for *Myo2* three Eastern Palearctic alleles (B plus two derived allelic haplotypes) were separated by at least two substitutions from a larger cluster from all other regions (Fig 4B).

### Multi-locus phylogeny and divergence times

**Fig 5:**
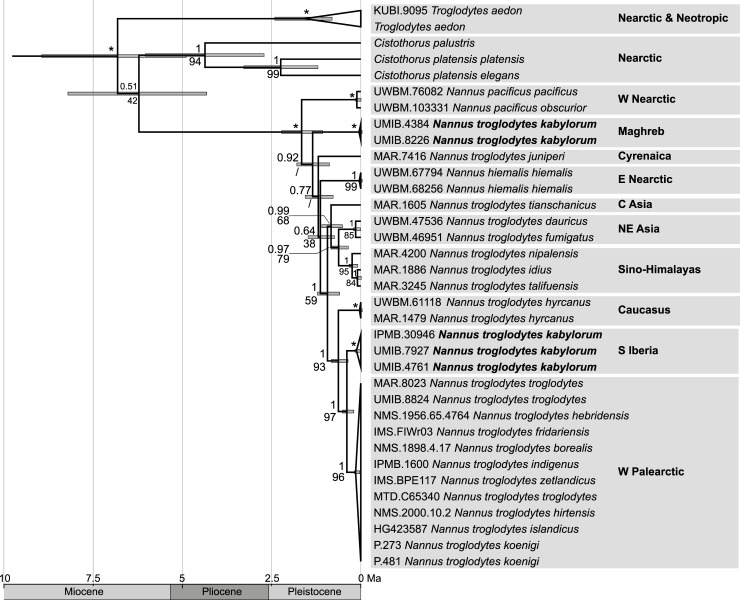
Multi-locus phylogeny of the *Nannus pacificus/hiemalis/troglodytes* species complex with outgroups reduced to *troglodytes aedon* and *Cistothorus* spp. (n = 35). Bayesian reconstruction across the five loci *COI* (696 bp), *ND2* (1033 bp), *Myo2* (736 bp), *Fib5* (570 bp), and *RAG1* (763 bp). Node support values indicate Bayesian posterior probabilities (above nodes) and bootstrap values of maximum likelihood analysis (below nodes; / = node was not recovered in the ML tree); asterisks mark nodes with full support from both analyses. Divergence times estimated with fossil calibration; 95% confidence intervals shown as node bars. Holocene (0.012–0 Ma, [81]) omitted on timescale for readability.

Our multi-locus phylogeny (Fig 5; see S2 Fig for all outgroup taxa included; S3 Fig for ML reconstruction) supported the monophyly of the *Nannus pacificus/hiemalis/troglodytes* species complex as the sister clade of *Troglodytes aedon* (1.00 PP). According to our time calibration, the differentiation of the *Nannus* species complex from its sister clade took place about 7.4 Ma ago in the late Miocene (late Tortonian).

According to the fossil time calibration of the multi-locus tree, the onset of diversification among the three *Nannus* wren species was dated to the early Pleistocene at about 2.0 Ma. Divergence times inferred from mtDNA substitution rates suggested two basically different scenarios. The maximum uncorrected mean rate for *ND2* was in good accordance with an early Pleistocene onset of the *Nannus* radiation (2.0–2.5 Ma) inferred from the fossil calibration (Fig 2B). Partitioning of mtDNA genes by codon position had little if any effect on tmrca estimates. However, the effect of body mass correction was considerable because divergence time estimates inferred from mass-corrected rates were almost twice as old as those inferred from the minimum uncorrected rate and from fossil calibration (Fig 2B). According to mass-corrected rates, the basal radiation of *Nannus* started already in the early Pliocene (~ 5.0 Ma). Tmrca estimates inferred from the minimum uncorrected rate supported the Pliocene diversification of *Nannus*. That scenario was consistent for nearly all runs with mass-corrected rates for the entire *ND2* gene and for the 3^rd^ codon position only (Fig 2B). Application of mass-corrected rates to the reduced Troglodytidae data set (all other families of Certhioidea and further outgroups excluded) yielded slightly younger divergence time estimates. This effect was strongest for mass-corrected rates for the 3^rd^ codon position that yielded a tmrca estimate for *Nannus* wrens at the Pliocene-Pleistocene boundary (Fig 2B).

In the Bayesian tree (Fig 5), the two Nearctic *Nannus* species (*N*. *hiemalis*, *N*. *pacificus*) and the two North African clades (*N*. *t*. *kabylorum* from the Western Maghreb and *N*. *t*. *juniperi* from the Cyrenaica) appeared as four deeply split successively basal offshoots of the *Nannus* clade. Neither the Nearctic species nor the two North African clades appeared as sister clades. However, none of the basal nodes received strong support, so the phylogenetic relationships of the four taxa in question were not fully resolved. In the ML tree *N*. *t*. *juniperi* from the Cyrenaica appeared as a basal offshoot and the two successively basal splits were i) a clade uniting Maghrebian *N*. *t*. *kabylorum* and Nearctic *N*. *pacificus* with poor support for this sister group relationship, and ii) Nearctic *N*. *hiemalis* (S3 Fig).

The terminal clade comprising all samples of continental Eurasian *N*. *troglodytes* received full support in both BI and ML analysis (Figs 5 and S3). That Eurasian clade was divided in an Eastern and a Western Palearctic subclade that were both strongly supported. East-West divergence among the two Eurasian continental subspecies groups was dated to Pleistocene in all time calibrations (e.g. to about 0.9 Ma based on the fossil-calibration, to 1.2–1.3 Ma based on uncorrected *ND2* rate, and to slightly earlier periods at the Pliocene-Pleistocene boundary (2.8 Ma) based on mass-corrected rates. Each of the two Eurasian subclades comprised three major genetic lineages (Fig 5). In the Western Palearctic these are i) *N*. *t*. *hyrcanus* from the Caucasus that was sister to a terminal sister group of ii) *N*. *t*. *kabylorum* from southern Iberia and iii) the large nominate clade from continental Europe (nominate *N*. *t*. *troglodytes*), the British Isles (sspp. *zetlandicus*, *fridariensis*, *hebridensis*, *hirtensis*, *indigenus*), other Atlantic Islands (sspp. *islandicus*, *borealis*) and Corsica-Sardinia (ssp. *koenigi*). In the Eastern Palearctic three distinct clades are represented by i) *N*. *t*. *tianschanicus* from Central Asia that was sister to a terminal sister group of ii) populations from the Sino-Himalayas (sspp. *nipalensis*, *talifuensis*, *idius*) and iii) populations from the Russian Far East and Japan (sspp. *dauricus*, *fumigatus*).

### Species distribution model for Western Palearctic *Nannus troglodytes*

**Fig 6:**
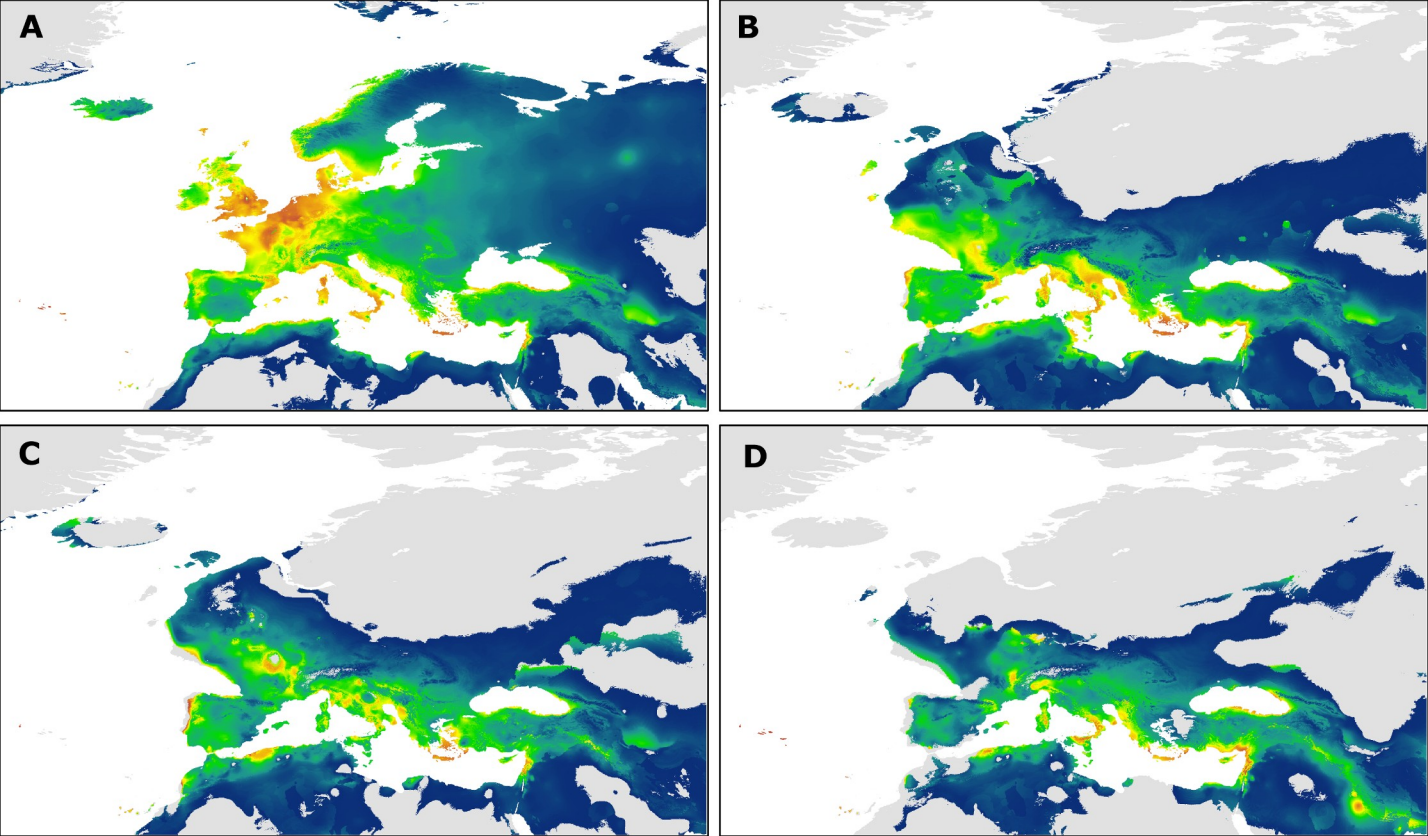
Results of species distribution modelling for Western Palearctic *Nannus troglodytes*. Current potential distribution as derived from Maxent (A). Projection onto climatic conditions of the Last Glacial Maximum as derived from the global circulation models MPI-ESM-P (B), CCSM3 (C), and MIROC-ESM (D). Suitability ranges from moderate (dark blue) to high (red).

The SDM for the current distribution of *N*. *troglodytes* in the Western Palearctic as well as the reconstructed SDMs for distributions during the LGM are shown in Fig 6. Model performance across all replicate runs was high for current (AUC test = 0.842) as well as past climatic conditions (CCSM3 = 0.838; MPI-ESM-P = 0.83; MIROC = 0.841) indicating that the model discriminates well between suitable and unsuitable space. Across all models, temperature seasonality (bio 4) had the highest contribution (31.4–34.1%), followed by precipitation of the driest quarter (bio 17, 21.4–21.4%), precipitation seasonality (bio 15, 17.7%), mean diurnal temperature range (bio 2, 8.2%), and maximum temperature of the warmest month (bio 5, 7.6%). Contribution of the remaining predictors did not exceed 5%. For details on variable contributions of all models see Table 1.

**Table 1:** Contribution of selected environmental predictor variables. Variable contributions exceeding 5% are displayed in bold; temperature abbreviated as T.

Predictor	Predictor name	Variable contribution (%)			
		current	CCSM3	MPI-ESM-P	MIROC
**Bio 2**	Mean diurnal T range	**8.2**	**8.9**	**9.9**	**7.5**
**Bio 3**	Isothermality	3.4	3.8	3.6	4
**Bio 4**	T seasonality	**34.1**	**32.9**	**31.8**	**32.2**
**Bio 5**	Max. T of the warmest month	**7.6**	**9.0**	**9.6**	**8.5**
**Bio 8**	Mean T of the wettest quarter	2.7	2.8	2.4	3.5
**Bio 12**	Annual precipitation	4.8	3.8	4.1	4.9
**Bio 15**	Precipitation seasonality	**17.8**	**16.5**	**13.7**	**17.3**
**Bio 17**	Precipitation of the driest quarter	**21.4**	**22.3**	**24.8**	**22.1**
**Total**		**100**	**100**	**100**	**100**

A comparison of the SDM for current and LGM conditions reveals that the extent of suitable space was considerably smaller during the LGM (Fig 6). Areas of high suitability indicate that the species range in the Western Palearctic could have contracted to isolated refugia situated in the Mediterranean region. A larger central refuge (or chain of smaller refuges) existed on the Italian Peninsula, the Balkan Peninsula, and some islands in the South of the continent (Corsica, Sardinia, Sicily, and Crete; Fig 6B–6D). Potential Iberian refugia could have existed rather in the West of the peninsula, whereas in the Eastern Mediterranean, small glacial refugia may have existed in the Levant and on Cyprus (Fig 6B–6D). In North Africa scattered glacial refugia might have existed in the Maghreb and these were largely separated from a small isolated refuge in the Cyrenaica according to all three models (Fig 6B–6D).

## Discussion

According to our phylogenetic analysis, genetic diversification of *Nannus troglodytes* is considerably greater than previously documented. In addition to four known mitochondrial lineages on the Eurasian continent (Europe, Caucasus, Nepal, and Eastern Asia [35]) we identified further four distinct clades of the *N*. *troglodytes* phylogeny: two in the Ibero-Maghrebian region (two clades of *N*. *t*. *kabylorum*), one in the Cyrenaica (*N*. *t*. *juniperi*), and one in Central Asia (*N*. *t*. *tianschanicus*). Monophyly of *N*. *troglodytes* was not supported by our multi-locus analysis and received only poor support from the single-locus analysis by Drovetski *et al*. ([35]; who excluded the two North African clades that rendered *N*. *troglodytes* paraphyletic). The two Nearctic species *N*. *pacificus* and *N*. *hiemalis* did not result as sister clades either (this study; [35]); yet there are also examples for non-monophyletic Nearctic species groups from other Holarctic passerine genera, e.g. kinglets, *Regulus* [117], and nuthatches, *Sitta* [118]. However, in wrens, none of the three basal nodes of the *Nannus* clade received reasonable support. Therefore, no firm conclusions on colonization pathways between the Nearctic and the Palearctic can be drawn from this poorly supported topology. Drovetski *et al*. [35] postulated five Pleistocene vicariant events that triggered diversification of *Nannus* wrens into six clades from a single Holarctic ancestor. In accordance with Drovetski *et al*. [35], our divergence time estimates inferred from fossil dating suggested that *Nannus* wrens started diversifying in the early Pleistocene when Nearctic, Eurasian and North African lineages separated from each other during a very short time period (2.0–1.2 Ma). We advocate for the Pleistocene scenario of *Nannus* wren diversification as the more reliable one, because it is supported by i) tmrca estimates based on the maximum uncorrected *ND2* rate for passerine birds [94] whereas the minimum uncorrected *ND2* rate was based on a mixed data set of Passeriformes and non-Passeriformes [96,119] and would thus be expected to pre-date passerine lineage splits to implausibly older ages; ii) tmrca estimates based on the mass-corrected *ND2* rate for the *Nannus* data set only (molecular clock calibration based on mass-corrected rates should rely on data sets of closely related species of comparable body mass [99]).

Though most tmrca estimates based on mass-corrected substitution rates for *ND2* suggested a more ancient (Pliocene) basal split of *Nannus*, early diversification on the Eurasian continent (basal split of the terminal crown clade) was unanimously dated to the Pleistocene in all our calibrations (see below). In the Eastern Palearctic, three major clades diversified during the Pleistocene in a circum-Tibetan phylogeographic pattern, that has been documented for many other passerine birds [120–122]: i) a Central Asian clade, ii) a Far East Russian/Japanese clade, and iii) a Sino-Himalayan clade. In the latter, the split between *N*. *t*. *nipalensis* from Nepal vs. Chinese *N*. *t*. *idius* and *N*. *t*. *talifuensis* corresponds to a characteristic east-west disjunction found in many other birds as well [120,121,123].

In the Western Palearctic however, the most striking diversification patterns were observed in the Ibero-Maghrebian and Cyrenaican regions.

### Phylogeographic patterns in the Ibero-Maghrebian and Cyrenaican regions

We could identify strongly divergent mtDNA lineages of Cyrenaican, Maghrebian, and Iberian wren populations. In our phylogeny, the two North African clades (*N*. *t*. *kabylorum* in the Maghreb and *N*. *t*. *juniperi* in the Cyrenaica) appeared as two early and deep splits from the *N*. *troglodytes* clade; however their phylogenetic relationships remained unresolved.

Biogeographical affinities between the Iberian Peninsula and North Africa are characterized by complex phylogeographical patterns and multiple phases of trans-Mediterranean bidirectional faunal exchange during Pliocene and Pleistocene times [12]. Strong genetic differentiation of Northwest African populations from closest Eurasian relatives as found in the Eurasian Wren is a common phylogeographic pattern in several other Palearctic bird taxa, such as *Strix aluco* [124–126], *Picus* spp. [127], *Pica* spp. [128], *Periparus ater* [129], *Cyanistes* spp. [27–29], *Acrocephalus scirpaceus* [130,131], *Certhia brachydactyla* [132], *Cinclus cinclus* [133], *Muscicapa striata* [134], *Ficedula* spp. [135,136], *Fringilla coelebs* [137,138], and *Loxia* spp. [139]. Similarly deep splits between Iberian and Maghrebian populations of the same species were found in other terrestrial vertebrates, such as in amphibians, reptiles [25,140], and in mammals [141–144]. The Strait of Gibraltar was suggested to be an effective biogeographic barrier for trans-Mediterranean floral and faunal interchange [145–147] and accordingly split ages between populations north and south of the Strait were often dated to times when land bridges between the two continents existed, e.g. during the Messinian Salinity Crisis (MSC) [23,148]. Earlier divergence times for the basal splits of Ibero-Maghrebian populations (*N*. *t*. *kabylorum* and *N*. *t*. *juniperi*) inferred from mass-corrected rates would be in accordance with that Pliocene scenario. There is even strong evidence for earlier Ibero-Maghrebian interchange prior to the MSC, e.g. in mammals [149], but also for more recent trans-Mediterranean faunal exchange including oversea-dispersal, stepping-stone colonization during phases of low sea-level [150,151] or even through human-mediated dispersal [152].

However, the entire Eurasian *Nannus* wren radiation was dated to the Pliocene-Pleistocene boundary and therefore the remarkable divergence between and within the East and West Palearctic clades was presumably shaped by range fragmentation and range shifts along with global climate cooling. Accordingly, phylogeographic patterns on the Eurasian continent, in the Mediterranean Basin, and in North Africa coincide with major glacial refugia as identified in our SDM analysis (i.e. the southern European peninsulas [4,153]).

In North Africa, Pleistocene origin of an east-west disjunction as suggested for *N*. *troglodytes* was suggested for several other vertebrate species, such as in reptiles (e.g. in chelonians: *Testudo graeca* [154]; and in lizards: *Acanthodactylus pardalis* group [155]), in birds (e.g. *Cyanistes teneriffae* [27–29], *Galerida cristata* [156], and *Fringilla coelebs* [30]) and in mammals (e.g. *Jaculus orientalis* [157]). Rather complex phylogeographic patterns have been documented for other mammals, e.g. vicariance of genetically distinct Red Fox populations (*Vulpes vulpes*) of the Maghreb and the Fertile Crescent that are replaced by Rueppell’s Fox (*Vulpes rueppellii*) across their distribution gap in Libya [143]. In other mammal species, Sardinian and other Mediterranean island populations are firmly nested in a trans-Maghrebian clade (e.g. in the European Wildcat, *Felis silvestris* [144]), or in a distinctive East Maghrebian clade (e.g. in the Greater White-toothed Shrew, *Crocidura russula* [158]).

Even if we considered an earlier onset of *Nannus* radiation (as inferred from mass-corrected *ND2* rates) we can assume that North African wren populations were affected by environmental changes along with the global cooling towards the end of the Pliocene and during the Pleistocene, e.g. during phases of aridification in North Africa at 2.8 Ma and 1.7 Ma [159,160]. As the establishment of Pleistocene glacial/interglacial climatic cycles resulted in the alternation of humid- and arid-adapted vegetation in the Mediterranean Basin [159–161], wrens might have occupied a broader range along the North African coastline during a relatively humid phase providing suitable bridging habitats and then have retreated into the Maghrebian Atlas Mountains and the Cyrenaican Jebel Akhdar massif due to the onset of increasing lowland aridification. Paleoenvironmental studies suggested that during the late Pleistocene the Jebel Akhdar constituted an “environmental refugium” from the extreme arid conditions in the neighbouring Sahara [162–164]. According to all three models compared in our SDM analysis, suitable habitats for *N*. *troglodytes* in the Maghreb and in the Cyrenaica were separated by a large corridor of unsuitable habitat (clearest separation according to CCSM3 and MIROC-ESM; Fig 6C and 6D). Thus, the extant distribution of *N*. *troglodytes* in North Africa well corresponds to a postulated Mauritanian and a Cyrenaican refugium [12].

### Cryptic lineages in Western Palearctic wrens

The remaining *N*. *troglodytes* taxa of the Eurasian continent showed a typical differentiation into Western and Eastern Palearctic sister clades, which is a common differentiation pattern of Palearctic birds observed on different taxonomic levels, such as within species (e.g. in tits [165] and in magpies [128]) or between closely related species pairs (e.g. in corvids [166] and in buntings [167]).

Within the Western Palearctic clade of Eurasian Wrens, we identified the Caucasian clade of *N*. *t*. *hyrcanus* as a first basal offshoot. According to DNA barcoding, there is first evidence that *N*. *t*. *cypriotes* of the eastern Mediterranean (distributed on Aegean Islands, western and southern Anatolia, Cyprus, and Levantine coastline [38]), is closely related to the Caucasian lineage (S1 Fig) [168]. However, the existence of another distinct mtDNA lineage (of *N*. *t*. *cypriotes*) in the eastern Mediterranean Basin needs further support from future analyses based on a broader sampling. Further potential glacial refugia in the eastern Mediterranean and the Middle East identified by our SDM analyses could have harboured ancestors of extant *N*. *t*. *cypriotes* and *N*. *t*. *hyrcanus* (Fig 6).

In a terminal late Pleistocene colonization event, founder populations from the European continent spread to the North Atlantic islands [49,50]. According to our SDM analyses suitable glacial refugia in south-western Europe could have harboured founder populations that colonized the British Islands via land bridges during the LGM (Fig 6B–6D).

On the Iberian Peninsula, we found a north-south disjunction between southern Iberian populations of *N*. *t*. *kabylorum* that represent a cryptic phylogenetic lineage and northern populations of the nominate ssp. *troglodytes* that were nested in the European nominate clade. Strong genetic divergence of two mtDNA lineages on the Iberian Peninsula was also observed in many plant and animal species (reviewed in [4,10]), including e.g. amphibians [140,169], mammals [170], and bird species such as the White-throated Dipper, *Cinclus cinclus* [133,171,172] and Savi’s Warbler, *Locustella luscinoides* [173]. Particularly, in the latter species local admixture of two divergent mtDNA lineages in Iberian population is striking; however, due to low sample sizes on the Iberian Peninsula in our study we cannot infer reliable information on levels of mitochondrial introgression. This must be subject to future population genetic analyses based on a comprehensive sampling of Iberian populations.

The Iberian Peninsula is regarded as one major Pleistocene glacial refugium in the Western Palearctic and as an important origin of post-glacial expansion of species to Central Europe. According to our SDM analysis isolated areas of suitable habitat during the LGM existed only along the west coast of the Iberian Peninsula. Ancestors of the extant genetically distinct *N*. *t*. *kabylorum* populations from southern Spain might have survived the Pleistocene in that region.

Along the southern European coastline a long chain of suitable habitat for *N*. *troglodytes* has existed during the LGM (Fig 6B–6D), e.g. in two other classical European glacial refugia (on the Apennine Peninsula and on the Balkan Peninsula). These central Mediterranean refugia must have been occupied by ancestral populations of the nominate *troglodytes* clade who later re-colonized large parts of Europe (including the Atlantic islands and the northern parts of the Iberian Peninsula) in one post-glacial expansion event. Very likely, Holocene range expansion of *N*. *t*. *troglodytes* to northern Iberia has limited the northward dispersal of the Iberian relict populations (i.e. the cryptic ssp. *kabylorum* lineage from southern Iberia) and shaped the intra-Iberian differentiation we observe today.

A similar pattern of post-glacial range expansion has been inferred for the Dunnock, *Prunella modularis* [174]. In this species, northern Europe appears to have been colonised from Apennine and Balkan refugia, rather than from genetically distinct Iberian or Caucasian lineages. Considering the heterogeneous distribution of wrens in Iberia with a continuous distribution in the north and rather scattered distribution in the southern part [175,176], this distributional pattern might already reflect genetic differentiation of wren lineages. Whether a zone of sympatry exists between the ranges of the cryptic south Iberian “*kabylorum*”-populations and north Iberian *troglodytes*-populations, is not known to date and would be subject to more detailed future research.

## Supporting information

S1 Fig TCS haplotype network drawn for the mitochondrial barcoding marker *COI* (551 bp) of the *Nannus pacificus/hiemalis/troglodytes* species complex. For n = 81 sequences, including 18 subspecies of *N*. *troglodytes*. Haplotype circles scaled to sample size (n) of each represented haplotype; substitution distances not to scale.
(JPG)

S2 Fig Multi-locus phylogeny of the *Nannus pacificus/hiemalis/troglodytes* species complex with all outgroups included. Bayesian reconstruction across the five loci *COI*, *ND2*, *Myo2*, *Fib5*, and *RAG1*. Node support values indicate Bayesian posterior probabilities. Divergence times estimated with fossil calibration. 95% confidence intervals shown as node bars. Holocene (0.012–0 Ma, [87]) omitted on timescale for readability.
(JPG)

S3 Fig Maximum likelihood phylogeny as calculated with RAxML v.7.2.6. Node values indicate bootstrap support.
(JPG)

S1 Table Overview of newly sequenced samples of the *Nannus troglodytes/hiemalis/pacificus* species group and further outgroup taxa used for this study. GenBank accession numbers are given for each of the used markers (XXX = sequence data from own samples; accession numbers will be provided upon manuscript acceptance). Highlited samples (orange) were included in the multi-locus phylogenetic reconstruction; further samples (imported from GenBank) included in haplotype networks (*ND2* & *COI*) only. Geographic coordinates are given in parentheses if estimated. Abbreviations of collections and institutions (Inst.) for newly sequenced samples as follows (in alphabetical order): Burke Museum of Natural History and Culture (UWBM), University of Washington, Seattle, WA, USA; Institute of Medical Sciences (IMS), University of Aberdeen, Foresterhill, Aberdeen, UK; Institute of Pharmacy and Molecular Biotechnology (IPMB), Heidelberg University, Heidelberg, Germany; Muséum National d’Histoire Naturelle (MNHN), Paris, France; National Museums Scotland (NMS), Edinburgh, UK; Research Unit of Biodiversity (UMIB), Oviedo University, Mieres, Spain; Senckenberg Natural History Collections Dresden (SNSD), Museum of Zoology, Dresden, Germany; tissue collection of J. Martens (MAR).
(XLSX)

S2 Table Summary of primer pairs used for PCR and sequencing, together with respective PCR thermal cycle settings. (XLSX)
