# Correction: Phylogeny of the Eurasian Wren *Nannus troglodytes* (Aves: Passeriformes: Troglodytidae) reveals deep and complex diversification patterns of Ibero-Maghrebian and Cyrenaican populations

**DOI:** 10.1371/journal.pone.0238206

**Published:** 2020-08-25

**Authors:** The PLOS ONE Staff

## Notice of Republication

This article was republished on July 20, 2020, to correct Fig 2. The publisher apologizes for the error. Please download this article again to view the correct version. The originally published, uncorrected article and the republished, corrected articles are provided here for reference.

## Supporting information

S1 File Originally published, uncorrected article. (PDF)

S2 File Republished, corrected article. (PDF)
