# Supplementary figures and images for: Phylogeny of the Eurasian Wren Nannus troglodytes (Aves: Passeriformes: Troglodytidae) reveals deep and complex diversification patterns of Ibero-Maghrebian and Cyrenaican populations
Source: PLoS One. 2020 Mar 19;15(3):e0230151. doi: 10.1371/journal.pone.0230151 (PMC7082076; doi:10.1371/journal.pone.0230151)

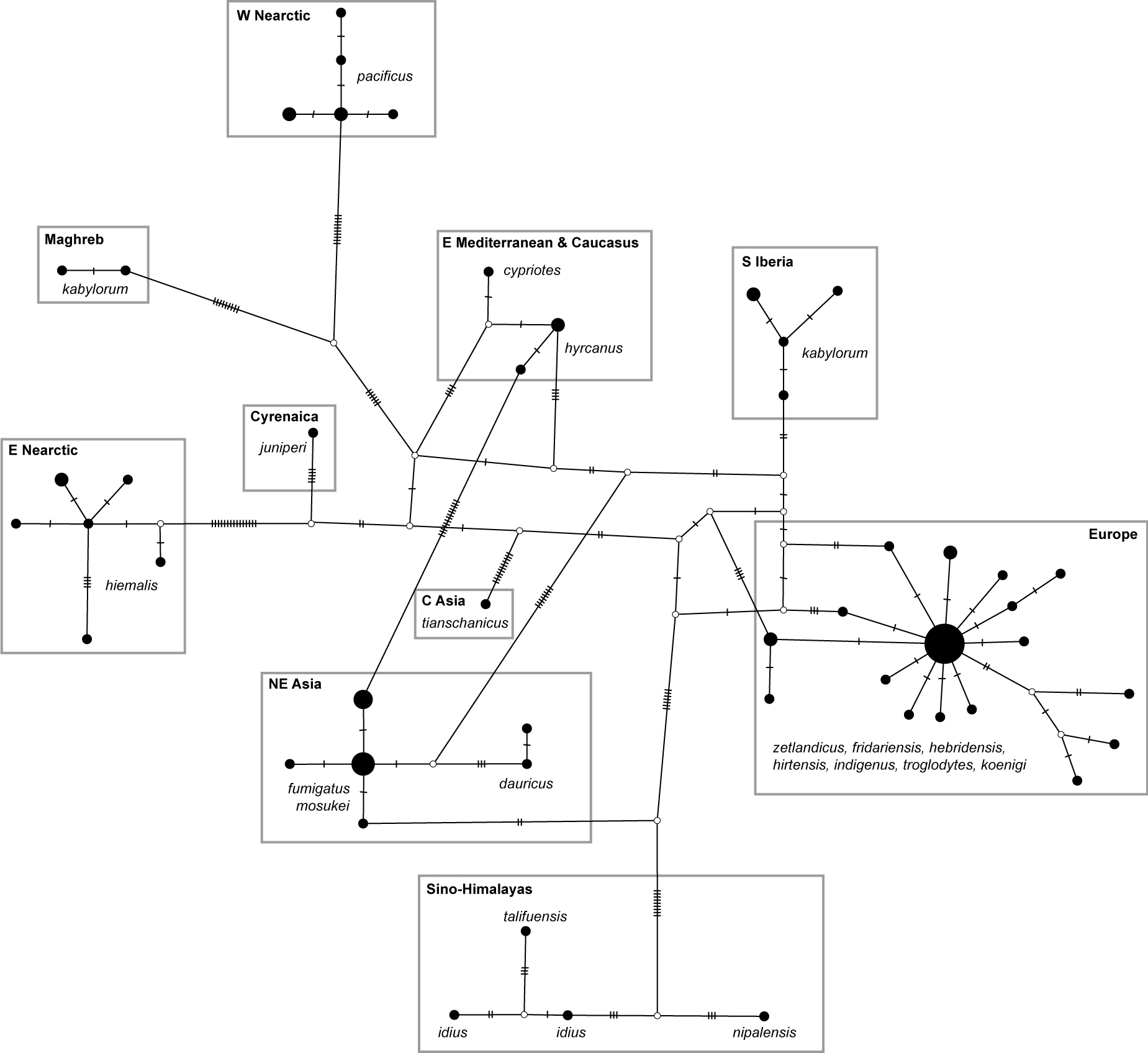

Supplement: S1 Fig — For n = 81 sequences, including 18 subspecies of N. troglodytes. Haplotype circles scaled to sample size (n) of each represented haplotype; substitution distances not to scale. (JPG) [file pone.0230151.s001.jpg]

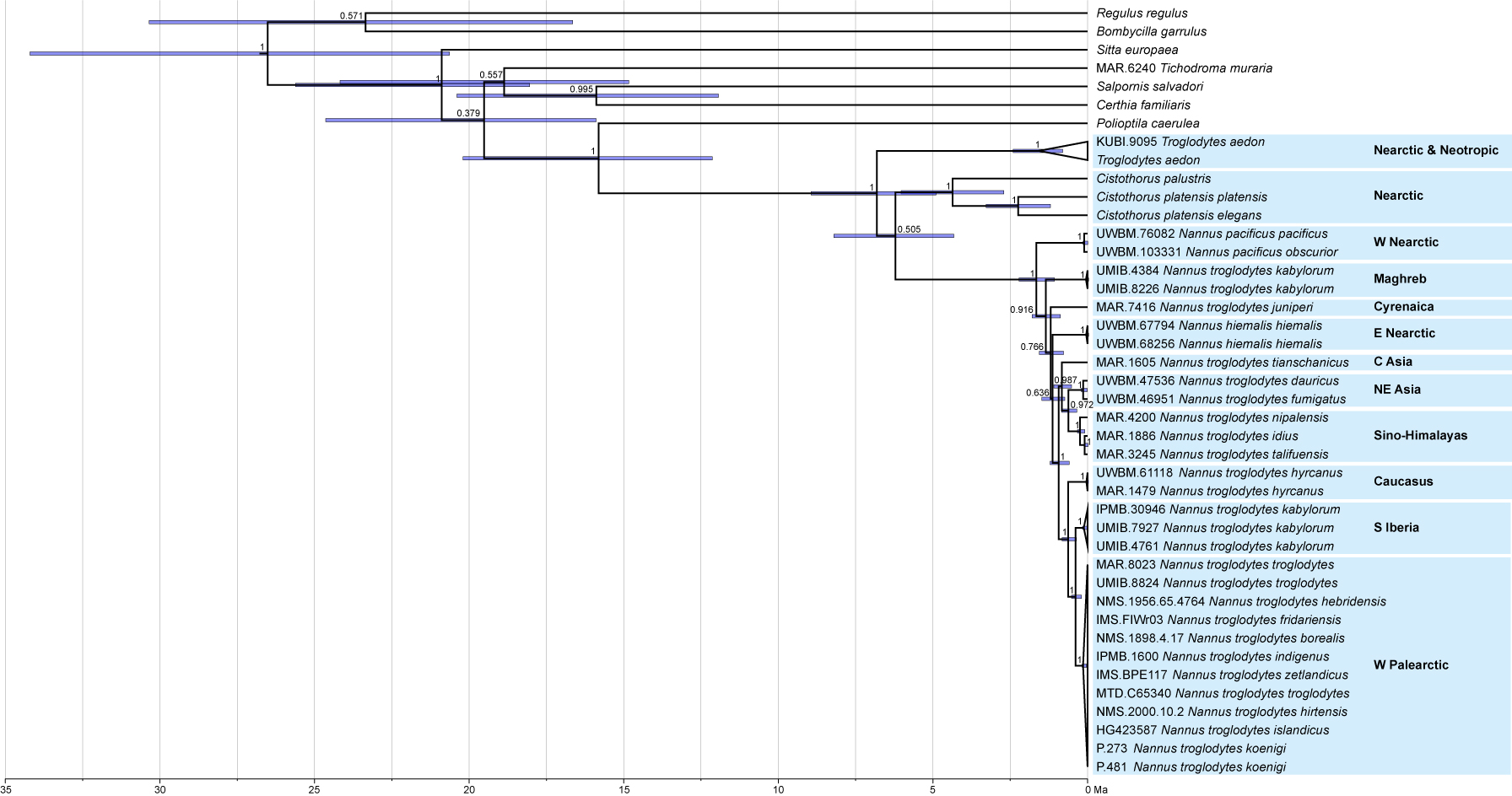

Supplement: S2 Fig — Bayesian reconstruction across the five loci COI, ND2, Myo2, Fib5, and RAG1. Node support values indicate Bayesian posterior probabilities. Divergence times estimated with fossil calibration. 95% confidence intervals shown as node bars. Holocene (0.012–0 Ma, [87]) omitted on timescale for readability. (JPG) [file pone.0230151.s002.jpg]

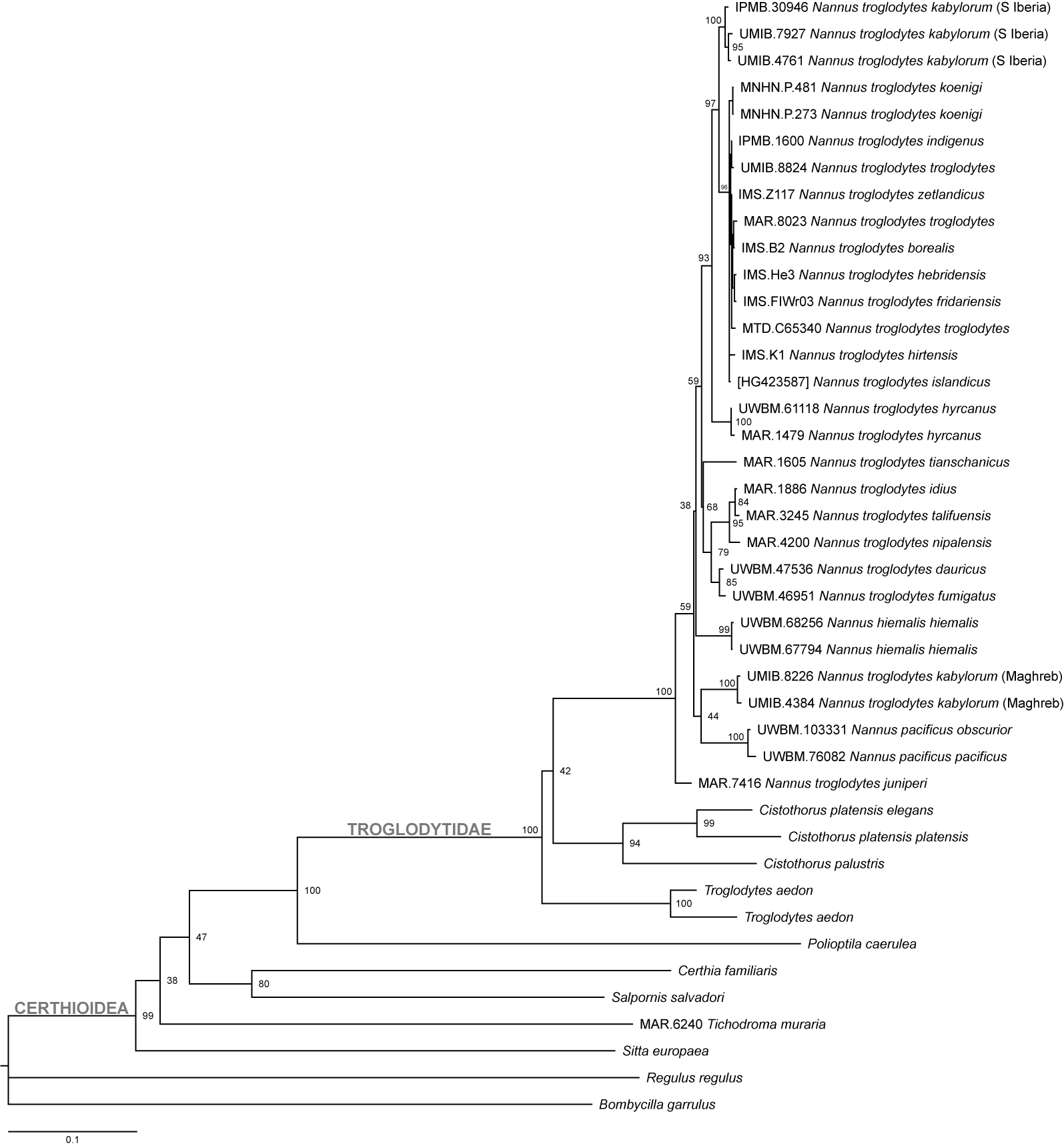

Supplement: S3 Fig — Node values indicate bootstrap support. (JPG) [file pone.0230151.s003.jpg]
