# Supplementary material for: Correction: Phylogeny of the Eurasian Wren Nannus troglodytes (Aves: Passeriformes: Troglodytidae) reveals deep and complex diversification patterns of Ibero-Maghrebian and Cyrenaican populations
Source: PLoS One. 2020 Aug 25;15(8):e0238206. doi: 10.1371/journal.pone.0238206 (PMC7446969; doi:10.1371/journal.pone.0238206)
Supplement: S2 File — (PDF) [file pone.0238206.s002.pdf]

PLOS ONE

RESEARCH ARTICLE

Phylogeny of the Eurasian Wren *Nannus troglodytes* (Aves: Passeriformes: Troglodytidae) reveals deep and complex diversification patterns of Ibero-Maghrebian and Cyrenaican populations

Frederik Albrecht[1]*, Jens Hering[2], Elmar Fuchs[3], Juan Carlos Illera[4], Flora Ihlow[1], Thomas J. Shannon[5], J. Martin Collinson[5], Michael Wink[6], Jochen Martens[7], Martin Päckert[1]

1 Museum of Zoology, Senckenberg Natural History Collections Dresden, Senckenberg|Leibniz Institution for Biodiversity and Earth System Research, Dresden, Saxony, Germany, **2** Verein Sächsischer Ornithologen e. V., Limbach-Oberfrohna, Saxony, Germany, **3** Verein Sächsischer Ornithologen e.V., Weimar, Thuringia, Germany, **4** Research Unit of Biodiversity (UO-CSIC-PA), Oviedo University, Asturias, Spain, **5** School of Medicine, Medical Sciences and Nutrition, University of Aberdeen, Aberdeen, Scotland, United Kingdom, **6** Institute of Pharmacy and Molecular Biotechnology, Heidelberg University, Heidelberg, Baden-Württemberg, Germany, **7** Institute of Organismic and Molecular Evolution, Johannes Gutenberg University Mainz, Mainz, Rhineland-Palatinate, Germany

* frederik_albrecht@web.de

Abstract

The Mediterranean Basin represents a Global Biodiversity Hotspot where many organisms show high inter- and intraspecific differentiation. Extant phylogeographic patterns of terrestrial circum-Mediterranean faunas were mainly shaped through Pleistocene range shifts and range fragmentations due to retreat into different glacial refugia. Thus, several extant Mediterranean bird species have diversified by surviving glaciations in different hospitable refugia and subsequently expanded their distribution ranges during the Holocene. Such a scenario was also suggested for the Eurasian Wren (*Nannus troglodytes*) despite the lack of genetic data for most Mediterranean subspecies. Our phylogenetic multi-locus analysis comprised 18 out of 28 currently accepted subspecies of *N. troglodytes*, including all but one subspecies which are present in the Mediterranean Basin. The resulting phylogenetic reconstruction dated the onset of the entire Holarctic radiation of three *Nannus* species to the early Pleistocene. In the Eurasian Wren, two North African subspecies represented separate basal lineages from the Maghreb (*N. t. kabylorum*) and from the Libyan Cyrenaica (*N. t. juniperi*), being only distantly related to other Mediterranean populations. Although *N. troglodytes* appeared to be paraphyletic with respect to the Nearctic Winter Wren (*N. hiemalis*), respective nodes did not receive strong statistical support. In contrast, paraphyly of the Ibero-Maghrebian taxon *N. t. kabylorum* was strongly supported. Southern Iberian populations of *N. t. kabylorum* did not clade with Maghrebian populations of the same subspecies but formed a sister clade to a highly diverse European clade (including nominate *N. t. troglodytes* and eight further taxa). In accordance with a pattern also found in other birds, Eurasian

OPEN ACCESS

Citation: Albrecht F, Hering J, Fuchs E, Illera JC, Ihlow F, Shannon TJ, et al. (2020) Phylogeny of the Eurasian Wren *Nannus troglodytes* (Aves: Passeriformes: Troglodytidae) reveals deep and complex diversification patterns of Ibero-Maghrebian and Cyrenaican populations. PLoS ONE 15(3): e0230151. https://doi.org/10.1371/journal.pone.0230151

Editor: Łukasz Kajtoch, Institute of Systematics and Evolution of Animals Polish Academy of Sciences, POLAND

Received: August 1, 2019

Accepted: February 22, 2020

Published: March 19, 2020

Copyright: © 2020 Albrecht et al. This is an open access article distributed under the terms of the Creative Commons Attribution License, which permits unrestricted use, distribution, and reproduction in any medium, provided the original author and source are credited.

Data Availability Statement: All genetic sequences are available from the GenBank database (URL: https://www.ncbi.nlm.nih.gov/genbank/; accession numbers: MN919550-MN919644; MN927043-MN927084; MN931755-MN931781).

Funding: JCI was funded by a GRUPIN research grant from the Regional Government of Asturias, Spain (Ref.: IDI/2018/000151). JM gives his thanks

to the Feldbausch-Stiftung and Wagner-Stiftung at the Faculty of Biology of the Johannes Gutenberg University Mainz, Germany, for financial support. The publication of this article was funded by the Open Access Fund of the Leibniz Association. The funders had no role in study design, data collection and analysis, decision to publish, or preparation of the manuscript.

Competing interests: The authors have declared that no competing interests exist.

populations were split into a western clade (Europe, Caucasus) and an eastern clade (Central Asia, Sino-Himalayas, East Asia). This complex phylogeographic pattern revealed cryptic diversification in *N. troglodytes*, especially in the Iberio-Maghrebian region.

Introduction

In the Western Palearctic, the Mediterranean Basin represents a region of exceptional genetic and species diversity both of flora and fauna and it is therefore recognized as a Global Biodiversity Hotspot [1–5]. Due to the colonization of structurally very different peninsulas (Iberian, Apennine, Balkan, and Anatolian Peninsulas), island archipelagos (of the Mediterranean Sea and Macaronesia), and the North African coastline, many species occur in marginal or isolated distributional areas with regard to their European core distributions [3,6,7]. At a more narrow spatial scale, the Mediterranean Basin comprises multiple regional vegetational Biodiversity sub-Hotspots [8–10] which largely coincide with several climatically stable refugia, where many endemic species have survived during the Pliocene and Pleistocene [11–13].

Within the western Mediterranean Basin, the Ibero-Maghrebian Region (IMR) comprises the Iberian Peninsula and the Maghreb Region of Northwest Africa. The latter stretches from the northernmost part of the Western Sahara Territory over Morocco and northern Algeria to Tunisia [14,15]. The IMR has previously been described in different scientific contexts, such as seismotectonics [16–18] and biogeography [19–23]. It furthermore includes the three Biodiversity sub-Hotspots of the High and Middle Atlas, the Baetic–Rifan mountain complex, and Kabylia–Numidia–Kroumire and is neighboured by the Mediterranean Cyrenaica of northeast Libya [9,12]. Being part of the Mediterranean biome, the IMR is characterized by Mediterranean sclerophyllous forests, woodlands, and scrub, but also by continued Palearctic influence, both in floral and in faunal communities [3]. For examples, several Euro-Siberian tree and shrub species reach their southernmost range limits here in the form of Maghrebian exclaves (such as *Taxus baccata*, *Ilex aquifolium*, *Sorbus aria*, *Prunus avium*, *Populus tremula*, *Acer campestre* [6,24]). A commonly observed phylogeographical pattern in many vertebrate taxa of the IMR is a strong differentiation between Iberian and Maghrebian populations and in many cases, also a further differentiation of western and eastern Maghrebian populations (e.g. reviewed for amphibians and reptiles [15,25,26]). Further east in the Mediterranean Cyrenaica, small relict populations of bush- and forest-dwelling bird species are known to exist on the forested Jebel Akhdar massif. These are separated by a large desert area from their closest conspecifics in the Maghreb and therefore, some Cyrenaican populations represent distinct and relict genetic lineages (e.g. in African Blue Tits *Cyanistes teneriffae* [27–29] and in Common Chaffinches *Fringilla coelebs* [30]).

The Eurasian Wren *Nannus troglodytes* (until recently referred to as *Troglodytes troglodytes*; see explanation below) inhabits the IMR at the south-western periphery of its Palearctic-wide distributional range. Here, it prefers forest and shrubland habitats with dense undergrowth, often in the proximity of watercourses [31–34], such as forested stream valleys at higher elevations in North Africa [32]. It is the only Palearctic species of the otherwise Nearctic and Neotropic family of wrens (Passeriformes: Troglodytidae), which currently comprises up to 93 recognised species [31–33]. Conventionally, all Holarctic populations of the "Winter Wren" or "Northern Wren" had been united under the species-level taxon *Troglodytes troglodytes* [31,32,34], until Drovetski and colleagues [35] demonstrated that Palearctic and Nearctic populations are divided into separate mitochondrial DNA (mtDNA) lineages. As this genetic

divergence was paralleled by differences in territorial songs [36], a species-level split of Palearctic from Nearctic "Winter Wren" populations was recommended [37]. Today, most authorities accordingly recognize the Eurasian Wren *T. troglodytes* as an exclusively Palearctic species [33,38,39]. For its remaining Nearctic relatives, a high level of genetic divergence between eastern and western populations is accompanied by slight but consistent bioacoustic differentiation [40,41] and due to a lack of vocal admixture in an area of sympatry, reproductive isolation has been assumed [42]. Therefore, the Nearctic "Winter Wren" populations were furthermore split into two species: the Pacific Wren *T. pacificus* of the Western Nearctic and the Winter Wren *T. hiemalis* of the Eastern Nearctic [33,37,38].

According to a recent molecular study [43], there is increasing evidence that the genus *Troglodytes* (*sensu* e.g. [31–33]) is not monophyletic. Therefore it was recommended to transfer *T. troglodytes*, *T. pacificus*, and *T. hiemalis* into the re-established genus *Nannus* Billberg, 1828 (as already suggested before by [44,45]), and to restrict the genus name *Troglodytes* Vieillot, 1809, to a monophyletic group of New World taxa (see [46], as referenced in [38]). Although these taxonomic recommendations have not yet been implemented into a major taxonomic compendium, we assume that this will be inevitably the case in the near future and we therefore follow the suggestion by Barker [43] and henceforth refer to the spp. *troglodytes*, *pacificus*, and *hiemalis* as members of the genus *Nannus*.

The Eurasian Wren is highly polytypic and currently populations of *N. troglodytes* are assigned to 28 (the preferred concept throughout this study [38,39,47]) or to 29 [33] subspecies, showing the highest subspecies diversity in Europe (North Atlantic islands and Mediterranean Basin) and in Eastern Asia (China and northwest Pacific islands from Taiwan to Kamchatka). In both regions, a differentiation into island-endemic subspecies subtly differing in size-proportions, plumage-barring, and coloration can be observed [31,32,48].

Based on an analysis of mitochondrial *NADH dehydrogenase 2* (*ND2*), the taxonomic diversity of *N. troglodytes* corresponds to four separate mitochondrial lineages, comprising six subspecies so far documented by Drovetski *et al.* [35]: A European lineage (sspp. *troglodytes* and *indigenus*), a Caucasian (ssp. *hyrcanus*), a Nepalese (ssp. *nipalensis*), and an East Asian lineage (sspp. *dauricus* and *fumigatus*). Further molecular genetic analyses of North Atlantic island populations (sspp. *islandicus*, *borealis*, *zetlandicus*, *fridariensis*, *hirtensis*, *hebridensis*) found only small genetic divergence of these subspecies from populations of ssp. *indigenus* from Great Britain and Ireland and from continental European nominate *troglodytes*, suggesting recent differentiation [49,50]. All North Atlantic island subspecies thus originated from Palearctic rather than from Nearctic founders and did not substantially diverge since the colonization event [49]. Wren populations of the northernmost ranges in Europe and East Asia are migratory and leave their breeding grounds in winter, whereas remaining populations are largely sedentary to partially migratory [31,32].

Although previous studies [35,49,50] shed light on the intraspecific differentiation of the Eurasian Wren, a broader geographic and taxonomic coverage is much required. Also the phylogenetic relationships of *N. troglodytes* taxa distributed in the IMR within the Mediterranean Biodiversity Hotspot remain unknown. Due to their peripheral and fragmented range with regard to the Palearctic core distribution they might, however, yield important information on the biogeographic history of this species.

In this study, we aim to scrutinize the genetic structure of *N. troglodytes* populations based on a denser taxon sampling at the subspecies level. Our focus was set on the phylogenetic relationships of unstudied populations from the forested margins of the Qinghai-Tibet Plateau (i.e. sspp. *talifuensis* and *idius*) and of Ibero-Maghrebian wren populations, represented by the isolated and poorly investigated subspecies *N. t. juniperi* of the Cyrenaica [51–54], by *N. t. kabylorum* of Northwest Africa, the Balearic Islands, and southern Iberia, by *N. t. koenigi*

endemic to Corsica and Sardinia, and by the nominate form *N. t. troglodytes* of northern Iberia and the rest of the European mainland. As phylogeographic results based on mtDNA data alone do not necessarily need to be consistent with findings from nuclear DNA analyses [55], our phylogenetic analysis relied on two mitochondrial genes and three nuclear markers (two introns, one exon) for 18 out of the 28 subspecies, of which six subspecies have not been subject to genetic analysis to date.

Materials and methods

Sampling and laboratory procedures

We analysed a total of 45 tissue and blood samples from 35 localities in the Palearctic (Fig 1). For a list of used samples and GenBank accession numbers of newly analysed sequences (MN919550-MN919644; MN927043-MN927084; MN931755-MN931781) see S1 Table. Genetic sample material used for this study was obtained from the collections of natural history museums (NMS, MNHN, SNSD, UWBM). All sampling procedures are in compliance with animal research ethical guidelines of respective institutes, as well as with national guidelines of respective countries. Therefore, the study has not been formally approved by an animal research ethics committee. Material was specifically collected for this study i) in the UK (feathers lost during routine ringing operations licenced by the British Trust for Ornithology, thus not falling under animal care regulation; c.f. [49]), ii) in Spain, where field work was conducted under permits of the Council of Government of the Principality of Asturias (2013/001891) and of the Regional Government of Andalusia (ENSN/BRL//MCF). The dataset was completed with sequences of further ingroup as well as outgroup taxa archived in GenBank.

Total genomic DNA was extracted from ethanol- or buffer-preserved tissue and blood samples using an innuPREP DNA Mini Kit (Analytik Jena AG) or an innuPREP Blood DNA Mini Kit (Analytik Jena AG) respectively, following the manufacturer's protocol except for overnight incubation with proteinase K for cell lysis in both procedures. We also included DNA extracts from eight samples of North Atlantic island populations previously analysed by Shannon *et al.* [49].

For identification of mitochondrial lineages, we amplified and sequenced two mitochondrial genes: barcoding standard marker *cytochrome oxidase subunit I* (*COI*, 696 bp) [57] and *NADH dehydrogenase subunit 2* (*ND2*, 1033 bp). We sequenced *ND2* for 35 samples for comparison with sequence data sets available from previous studies (112 sequences; see below and S1 Table). To reconstruct a most taxon-complete multi-locus phylogeny of *Nannus* wrens, we sequenced three further nuclear markers for at least one sample of each subspecific taxon (or mitochondrial lineage per taxon): *beta-fibrinogen gene*, *intron 5* (*Fib5*, 570 bp), *myoglobin gene*,

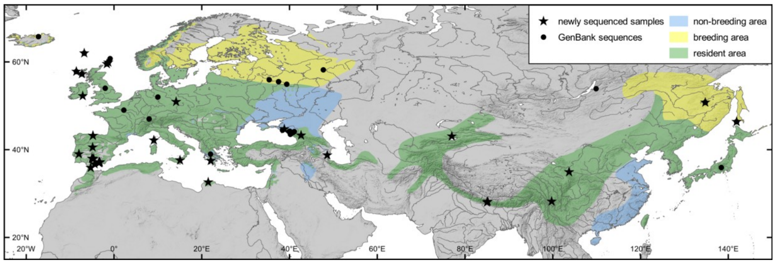

Fig 1. Distributional area of *Nannus troglodytes* in the Palearctic. Seasonal ranges indicated by colour (green: whole-year resident; yellow: breeding; blue: non-breeding; data from [56]), and genetic sampling localities for our phylogenetic analyses indicated by symbols (stars: newly sequenced samples for this study; circles: sequences retrieved from GenBank).

https://doi.org/10.1371/journal.pone.0230151.g001

intron 2 (*Myo2*, 736 bp), and one partial exon of the *recombination activating protein 1 gene* (*RAG1*, 763 bp) (see S2 Table for used primers and PCR protocols). To hydrolyse surplus primers and nucleotides, PCR products were purified using ExoSAP-IT (Thermo Fisher Scientific, Waltham, MA, USA) according to the manufacturer's instructions (adding 0.2 ml ExoSAP-IT solution in 4 ml H_2O to each sample; thermocycler incubation with 37˚C for 30 min, 94˚C for 15 min).

PCR products were prepared for sequencing with BigDyeTM 3.1 Dye Terminator Cycle Sequencing Kits (Applied Biosystems, now at Thermo Fisher Scientific, Waltham, MA, USA), according to the manufacturers' instructions. Cycle sequencing products were purified by using Sephadex (GE Healthcare, Munich, Germany), and sequenced in both reading directions on an ABI 3130xl DNA sequencer (Thermo Fisher Scientific, Waltham, MA, USA).

For each of the five markers, a sequence alignment was compiled in MEGA6 [58]: Forward and reverse DNA sequences for each individual sample were manually inspected and edited in a cross-check with the respective chromatogram signals (using Chromas v.2.6.5 (Technelysium Pty Ltd, Brisbane, Australia)). Sequences of both reading directions were then combined to a single consensus sequence per marker per sample. Further sequences of *Nannus* and *Troglodytes* taxa were imported from GenBank into the respective alignments to increase sample size and taxon coverage. For *ND2* and *COI* haplotype network reconstruction (see below), we included published sequences from GenBank into our datasets (see S1 Table). Furthermore, we included sequence data of at least one representative of all other families of Certhioidea (Sittidae, Certhiidae, Polioptidae) to the Troglodytidae data set (for full account of included sequences for phylogenetic analyses, see S1 Table). For hierarchical outgroup rooting we used sequence data of the Bohemian Waxwing (*Bombycilla garrulus*) and the Goldcrest (*Regulus regulus*).

Phylogenetic analyses

Single-locus analyses. For inference of phylogeographic structure, we reconstructed haplotype networks for all markers using PopART v.1.7 [59]. To determine allele sequences of nuclear markers in heterozygote individuals, we applied the PHASE algorithm as implemented in DNA Sequence Polymorphism v.6 (DnaSP 6 [60]), with MCMC options at their default values (number of iterations: 100; thinning interval: 1; burn-in iterations: 100). Allelic haplotype networks were then created as TCS networks [61,62], using PopART v.1.7 [59] with gaps being treated as a 5th state. For nuclear markers, we included sequences of *Troglodytes aedon* from GenBank as outgroups, as well as sequences of *Cistothorus palustris* and *C. platensis* published by Robbins and Nyári [63] for the marker *Fib5*.

Multi-locus analysis and divergence time estimates. We used Partitionfinder v.1.1.1 [64] to determine the best-fitting partitioning scheme and models of sequence evolution. The search for the best strategy was performed using the 'beast' model-set and heuristic search. The best fitting strategy according to the AICc criterion was a 9-partition scheme with both mitochondrial genes being split by codon positions and the three nuclear markers as separate partitions each (resulting partitioning scheme and corresponding substitution models: *COI*: 1st position: GTR+Γ+I, 2nd position: HKY+I, 3rd position: GTR+I; *ND2*: 1st position: TrN+Γ+I, 2nd position: GTR+Γ+I, 3rd position: GTR+Γ; *Fib5*: GTR+Γ+I; *Myo2*: HKY+Γ; *RAG1*: TrN+I).

A multi-locus tree was reconstructed using Bayesian inference of phylogeny in BEAST v.1.8.1 [65]. According to the best-fitting scheme, nine partitions were assigned to the five markers and the best-fitting models applied to each partition. All tree models were linked to one tree model and a 'Speciation: Birth-Death Incomplete Sampling' (BD model) tree prior was applied [66]. We optimized ESS values in exploratory runs with BEAST using different

chain lengths and priors. It turned out that robustness of the analysis was most strongly influenced by prior choice of the six rate parameters of the GTR model: We obtained poor ESS values across all parameters when a gamma distribution (default setting) was applied to rate priors; however, ESS values were greatly improved when a uniform prior distribution was applied. Tree-model choice did not affect tree topology at all in exploratory runs under the Yule model (compare [67,68]). Divergence time estimates were slightly older under the Yule model than under the BD model; however, 95% HPD intervals for the time of the most recent common ancestor (tmrca) largely overlapped among runs under different tree models (compare [69]).

MCMC chains ran for 50,000,000 generations in two parallel runs, each with one cold and three heated chains (heating parameter $\lambda = 0.1$). Trees were sampled every 5,000th generation. The first 3,000 samples were discarded as burn-in and model parameters and posterior probabilities were estimated from the remaining samples. Remaining trees were summarized in a 50% majority rule consensus tree. Effective sample sizes for priors used in the Bayesian inference of phylogeny were controlled in Tracer v.1.7.1 [70].The BEAST analysis was run three times using different seeds and convergence of BEAST runs was assessed after combining the three log files using LogCombiner v.1.7.1 into a single log file. The combined log file was inspected in Tracer v.1.7.1 and ESS values were checked for all parameters.

In addition, we reconstructed a multi-locus phylogeny using Maximum Likelihood (ML) with RAxML v.7.2.6 [71], using the GUI python application v.0.93 [72]. Node support in a ML framework was obtained by 1,000 bootstrap replicates with RAxML (thorough bootstrap option, 100 replicates). In two separate runs, we partitioned the concatenated matrix (5 partitions by gene; 9 partitions by gene and codon, see above) and applied the GTR+Γ+I model across partitions.

To estimate phylogenetic divergence times, we applied two methodological approaches in the form of i) molecular clock calibrations based on empirical substitution rates, and ii) a fossil time calibration. The latter was based on two fossil taxa of the superfamily Certhioidea (defined by [73]). Two fossils have been postulated as common ancestors of all Certhioidea, *Certhiops rummeli* Manegold, 2008 [74], from the early Miocene of Germany (MN3 20.5–18.0 Ma), and the recently described taxon *Kischinskinia scandens* Volkova & Zelenkov, 2018 [75], from the early Miocene of eastern Siberia (MN5 16.0–13.8 Ma [76]). The fossil age of the older fossil (*Certhiops*) was applied as estimate for the tmrca to the node uniting all Certhioidea. We performed our calibration according to the standard outlined by Benton *et al.* [77], who recommended the use of a soft maximum and minimum constraint that correspond to the oldest certain and the oldest possible date of origin of a clade. According to this approach, Claramunt and Cracraft [78] generated clade age priors for time calibration. Therefore we used their tmrca priors for Certhioidea for our fossil calibration: zero offset = 18.0; Log(mean) = 2.0; Log(stdev) = 1.2 (thus the known fossil age 20.5–18.0 Ma covered the time interval from the zero offset to the maximum of the lognormal prior distribution (c.f. [77]: Fig 2; [78]: Fig 1). Because monophyly of Certhioidea was strongly supported in previous analyses [79,80], the calibrated node was forced to be monophyletic. The geologic time scale applied in our analysis followed Walker *et al.* [81]. The trees obtained from the BEAST analysis were summarised to one consensus tree applying TreeAnnotator v1.8.1, using a burn-in set to a number of 3,000 trees and mean node heights. The final consensus trees were visualised in FigTree v.1.4.3 [82] with posterior probabilities (PP) and ML bootstrap as node support values.

For comparison, we inferred divergence time estimates based on empirical substitution rates for *ND2* from multiple independent runs with BEAST under a relaxed lognormal clock model. Some previous studies have considered the empirical substitution rate for the avian cytochrome-*b* (0.0105 substitutions per site per lineage per Ma [83]) as a 'universal' value for

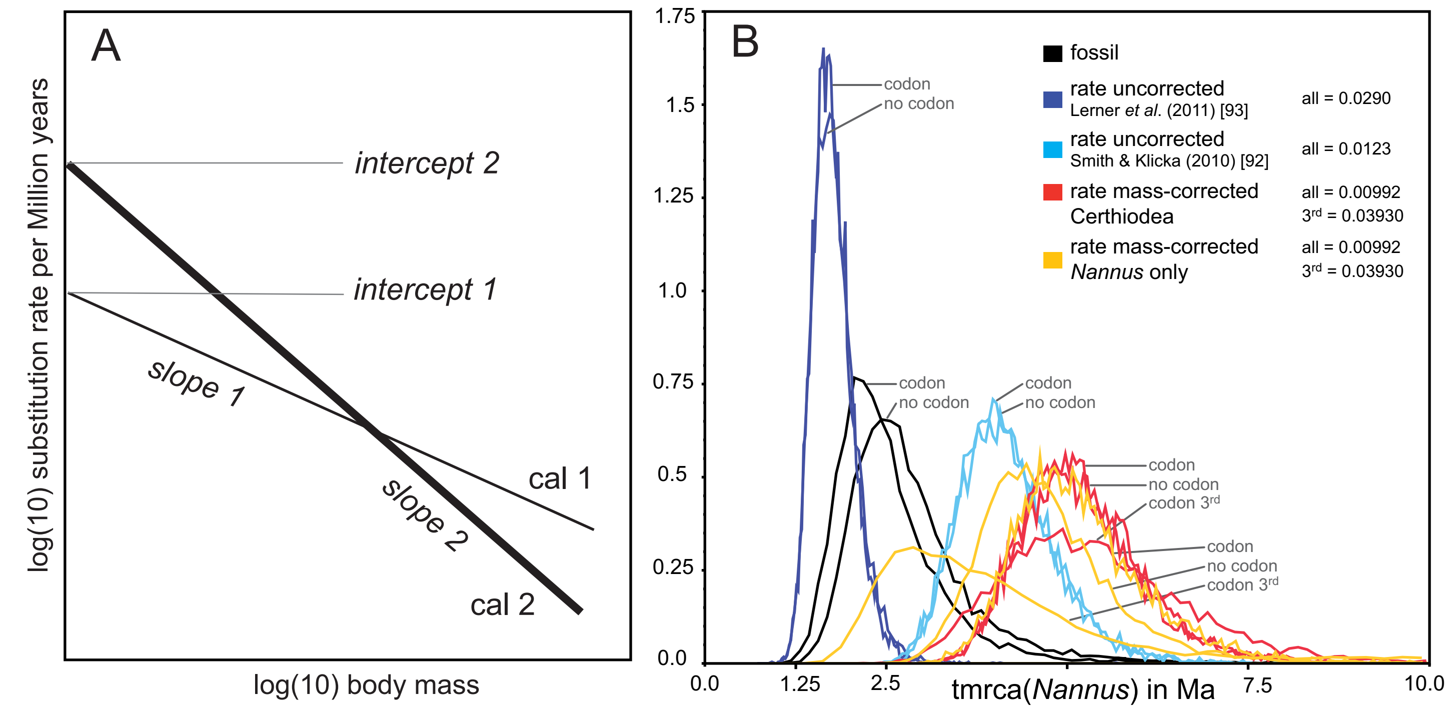

Fig 2. Inference of divergence time estimates from mitochondrial substitution rates. A: linear correlation between body mass and mitochondrial substitution rates in birds according to two calibration sets (cal1, cal2) by Nabholz *et al.* [99]; mass-corrected rates can be inferred from two parameters of the regression lines: slope and intercept; B: divergence time estimates for the time of the most recent common ancestor (tmrca) of *Nannus* according to 12 independent runs with BEAST; marginal density plots inferred from log output files with Tracer v.1.7.1; divergence time estimates were inferred from i) fossil calibration (by gene: *ND2* treated as a single partition; by codon: *ND2* partitioned by codon position), ii) two uncorrected rates for *ND2* (from [92] and [93], each partitioned by gene or by codon; see above), iii) a mass-corrected rate for the entire *ND2* gene of *Nannus* (partitioned by gene or by codon; see above), and iv) a mass corrected rate for the 3rd codon position of *ND2* in *Nannus* (partitioned by codon; see above); rates in substitutions per site per lineage per Ma indicated at upper left.

https://doi.org/10.1371/journal.pone.0230151.g002

mtDNA in general [84]. However, this approach is problematic as substitution rates vary greatly among mitochondrial genes (birds: [85–87]; other vertebrates: [88,89]) and the substitution rate of a selected mtDNA marker may vary drastically among different bird orders with passerine mtDNA evolving fastest [90–92]. Divergence time estimates for *Nannus* wrens were previously inferred using an empirical rate based on a molecular clock calibration for *ND2* of Galapagos mockingbirds of 0.0276 substitutions per site per lineage per million years [35]. However, empirical rate estimates for *ND2* cover a rather broad range from 0.0123 to 0.029 subst/site/lin/Ma [93–96]. We therefore performed independent runs with BEAST based on the latter empirical maximum and minimum estimates. Moreover, there is firm evidence that in birds mitochondrial substitution rates are correlated with body size, body mass, and generation time [97–99], so that systematic biases could arise if rates are not corrected for different species (but see [92] for a critical reappraisal). Because the Eurasian Wren is one of the smallest Palearctic birds, we expect a considerable deviation from empirical mean mtDNA substitution rates for this species. We therefore calculated body mass-corrected substitution rates for *ND2* of wrens according to the parameters inferred from two different calibration sets (2 and 4) by Nabholz *et al.* [99] (Fig 2). We assumed a mean body mass of *Nannus* wrens of 9 g [48]. We used mass-corrected rates of the entire *ND2* gene and for the 3rd codon position of *ND2* for further independent inference of divergence time estimates with BEAST. For reliable comparison with divergence time estimates based on the fossil calibration, we performed molecular clock calibrations using the entire Certhioidea data set of the *ND2* data set including all further outgroups. However, Nabholz *et al.* [99] recommended limiting data sets for molecular clock

calibration using mass-corrected rates to closely related species of comparable body mass values. Because many other Certhioidea species are considerably larger than wrens (e.g. several nuthatches), we performed alternative runs with BEAST using only the data set of Troglodytidae (with sequences of *Polioptila caerulea* for hierarchical outgroup rooting).

According to molecular clock calibrations based on calibration set 2 [99] time estimates for the most recent common ancestor (tmrca) of Certhioidea were nearly as old as the most ancient known stem fossil of all Passeriformes (tmrca of Certhioidea: 26–29 Ma; fossil age: 30–34 Ma, [100,101]). We do not consider this a reasonable result and one reliable explanation is that time calibrations relying on calibration points only for outgroup taxa or near the root of a phylogenetic tree tend to overestimate divergence times particularly for crown groups of a phylogeny [102,103]. We consider calibration set 4 more appropriate for passerine birds, as it included one further calibration point within Passeriformes that was not used in calibration set 2. Therefore, in the following we do not consider mass-corrected rates inferred from calibration set 2 and show only results inferred from calibration set 4 by Nabholz *et al.* [99].

Species Distribution Modelling (SDM)

To characterize the current distributional range of *Nannus troglodytes* based on its climatic niche and to draw conclusions on possible refugia of this species during the Last Glacial Maximum (LGM, ~ 22,000 years ago [104]), we performed species distribution models (SDMs) based on locality data covering the entire distributional range of this species and on a set of environmental predictor variables. Locality data was obtained in the form of geo-referenced collection data of preserved specimens from the online databases GBIF [105] and VertNet [106] (manually controlled for obvious mistakes, e.g. offshore occurrence, and corrected if possible), together with further occurrence data from the SNSD bird collection and type localities inferred from the literature [107]. Occurrence records based on geo-referenced tissue samples from earlier studies [35] and from the dataset compiled for this study (see Fig 1) were also included in the locality dataset. The dataset was spatially rarefied to a Euclidian distance of 5 km using the SDMtoolbox v1.1c [108] in ArcGIS v10.3 to prevent false inflation of model performance. Occurrences from assumed non-breeding wintering areas of partly migratory Eurasian Wrens in the Ponto–Caspian steppe (eastern Ukraine / southwest Russia) and in southeast China (Fig 1) were not included in the dataset.

As a result 583 unique records were retained for modelling. The following uncorrelated (correlation coefficients: $R^2 < 0.75$) bioclimatic variables with a spatial resolution of 2.5 arc-minutes (~4 km at the equator) describing annual trends, seasonality, and limiting factors related to temperature and precipitation, were obtained from http://worldclim.org [104]: mean diurnal temperature range (bio 2), isothermality (bio 3), temperature seasonality (bio 4), maximum temperature of the warmest month (bio 5), mean temperature of the wettest quarter (bio 8), annual precipitation (bio 12), precipitation seasonality (bio 15), and precipitation of the driest quarter (bio 17). To assess the influence of past climate and sea level fluctuations on the distribution of *N. troglodytes* corresponding predictor variables for three projections for the LGM, derived from global circulation models through the Paleoclimate Modelling Intercomparison Project Phase II [109], were obtained [104]. These include the Community Climate System Model (CCSM3) [110], the Max-Planck-Institute Earth System Model P (MPI-ESM-P), and the Model for Interdisciplinary Research on Climate (MIROC) [111].

A circular buffer of 400 km surrounding each locality was used as background for model training whereas a rectangular study extent was selected as projection area. Therefore, the model was trained across the entire breeding range of *N. troglodytes* but projections were restricted to a smaller extent focussing on the Western Palearctic (Europe + North Africa). By

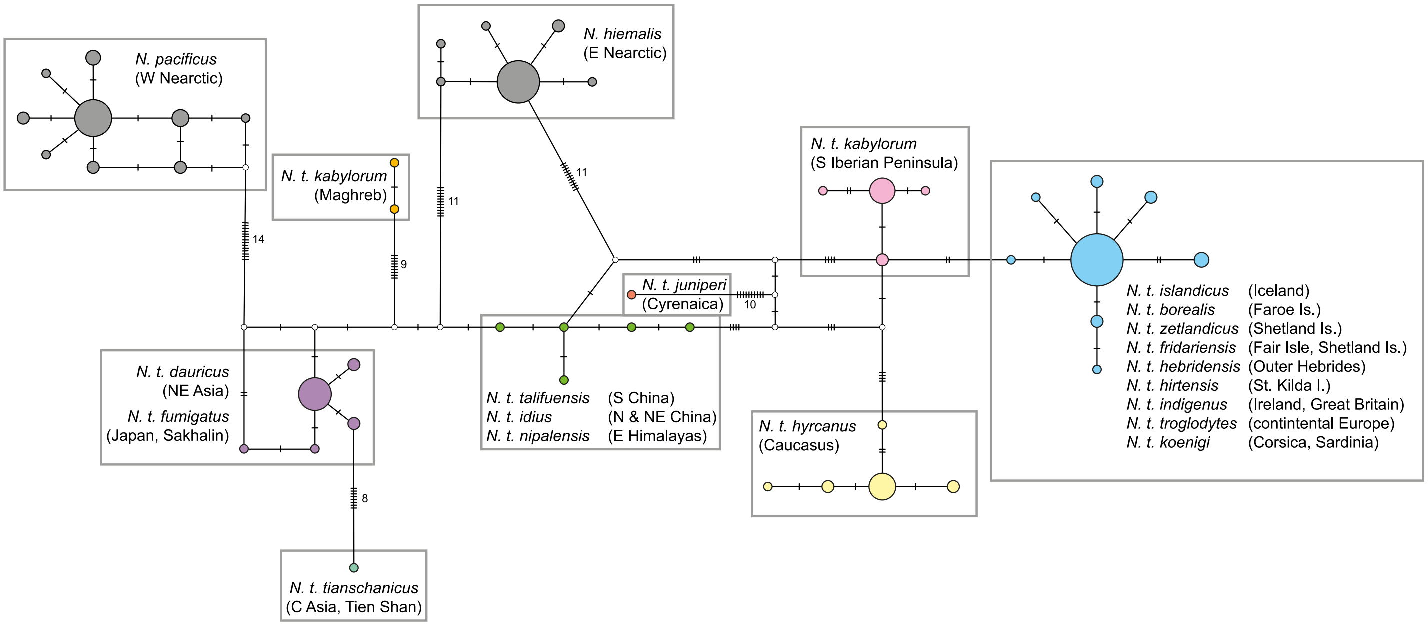

Fig 3. TCS haplotype network drawn for the mitochondrial *ND2* of the *Nannus pacificus/hiemalis/troglodytes* species complex including 18 subspecies of *N. troglodytes*. Haplotype circles scaled to sample size (n) of each represented haplotype (total sampling: n = 174); substitution distances not to scale.

https://doi.org/10.1371/journal.pone.0230151.g003

doing so, we accounted for a sampling bias between relatively many records in the Western Palearctic and relatively few records in the Eastern Palearctic. Models were computed using the machine learning algorithm Maxent v3.4.1 [112–114]. Maxent is one of the most efficient presence-only data modelling tools. The feature classes linear, quadratic, and hinge were selected. A bootstrapping method with 100 replicates randomly splitting the data set into a training (80%) and a testing subset (20%) was applied. Subsequently, the model was projected onto the three LGM projections. The area under the curve (AUC), a threshold-independent measure of model performance, was used for model evaluation [115]. An AUC score of 1 refers to a perfect fit of the data while a score of 0.5 is no better than random [112,116]. The average projection across all replicate runs was used for further processing, wherein the minimum training presence logistic threshold was applied as presence-absence threshold.

Results

Single-locus reconstructions

The haplotype network based on mitochondrial *ND2* sequences (Fig 3; n = 174, sequence length: 723 bp) included the highest number of polymorphic sites among all markers used for this study and showed a differentiation of sequences into 46 haplotypes. Within this network, the Nearctic species *Nannus pacificus* and *N. hiemalis* were found in two independent genetic clusters at different positions; both species are remarkably differentiated from any neighbouring Palearctic clusters. For the Palearctic *N. troglodytes*, eight haplotype clusters could be identified as listed below:

1. **Widespread Western Palearctic.** This haplotype cluster comprised most of the Western Palearctic samples and included the nominate subspecies *N. t. troglodytes* together with seven insular subspecies from the North Atlantic Ocean (sspp. *islandicus*, *borealis*, *zetlandicus*, *fridariensis*, *hebridensis*, *hirtensis*, *indigenus*) as well as the Mediterranean subspecies

koenigi (represented by two samples from Corsica sharing the same haplotype). The most common central haplotype (out of eight) of this cluster was found in 36 individuals from six subspecies. Three samples of nominate *N. t. troglodytes* from northern Spain (Picos de Europa) were also included in this cluster.

2. **Southern Iberia.** Samples of ssp. *kabylorum* from central and southern parts of the Iberian Peninsula (from Sotalbo, Province of Ávila, and from the Sierra Nevada, respectively) belonged to a separate mitochondrial cluster. This southern Iberian cluster comprised four haplotypes that differed from the central haplotype of the Western Palearctic cluster by at least three substitutions.

3. **Maghreb.** Strikingly, *N. t. kabylorum* populations from central and southern Iberia and those from Northwest Africa were not closely related; the two haplotype clusters differed by at least 20 substitutions.

4. **Cyrenaica.** The single haplotype of the isolated Cyrenaican population of *N. t. juniperi* did not fall into any other Mediterranean cluster but was placed in a separate cluster.

5. **Caucasus.** Further east on the Eurasian continent, individuals of *N. t. hyrcanus* from the Caucasus region formed a fifth cluster (of five haplotypes) that differed by at least seven substitutions from the Western Palearctic nominate cluster.

6. **Central Asia.** One haplotype of the Central Asian *N. t. tianschanicus* represented a sixth mitochondrial cluster that differed by at least eight substitutions from its nearest neighbouring cluster below.

7. **Northeast Asia.** Five haplotypes of the Northeast Asian and Japanese sspp. *dauricus* and *fumigatus* were assembled in this cluster. They differed by eight substitutions from the Central Asian haplotype of *N. t. tianschanicus* and by at least four substitutions from haplotypes of the Sino-Himalayan cluster.

8. **Sino-Himalayan.** This Asian cluster of the Sino-Himalayan region contained the haplotypes of Chinese sspp. *idius* (two haplotypes) and *talifuensis* (one haplotype), together with two Nepalese haplotypes of ssp. *nipalensis* at a distance of at least six substitutions.

A haplotype network based on the barcoding marker *COI* included 38 haplotypes of 18 subspecies of *N. troglodytes* which were allocated to the same clusters that were also identified by the *ND2* network (S1 Fig; n = 81, sequence length: 551 bp). Furthermore, the barcoding network included two samples which each represent an additional subspecies not included in the *ND2* haplotype network or in the multi-locus phylogeny: *N. t. cypriotes*, which was attributed to the cluster of *N.t. hyrcanus* from the Caucasus, and *N. t. mosukei* from the Izu Islands of Japan, sharing the most common haplotype within *N. t. fumigatus* (S1 Fig).

Allelic haplotype networks for the three nuclear markers are shown in Fig 4. All three markers showed a pronounced differentiation of *Troglodytes aedon* alleles from alleles of the *Nannus* species complex, including indels of several base pairs (bp) in all three cases. For *Fib5*, *Cistothorus* and *T. aedon* were separated by a shared indel of 47 bp from *Nannus* (Fig 4A). None of the nuclear markers showed a clear phylogeographic structure within *Nannus* and many alleles were shared among regions (e.g. *Fib5* allele B, *Myo2* alleles A and C, *RAG1* allele A; Fig 4). Only for *Myo2* three Eastern Palearctic alleles (B plus two derived allelic haplotypes) were separated by at least two substitutions from a larger cluster from all other regions (Fig 4B).

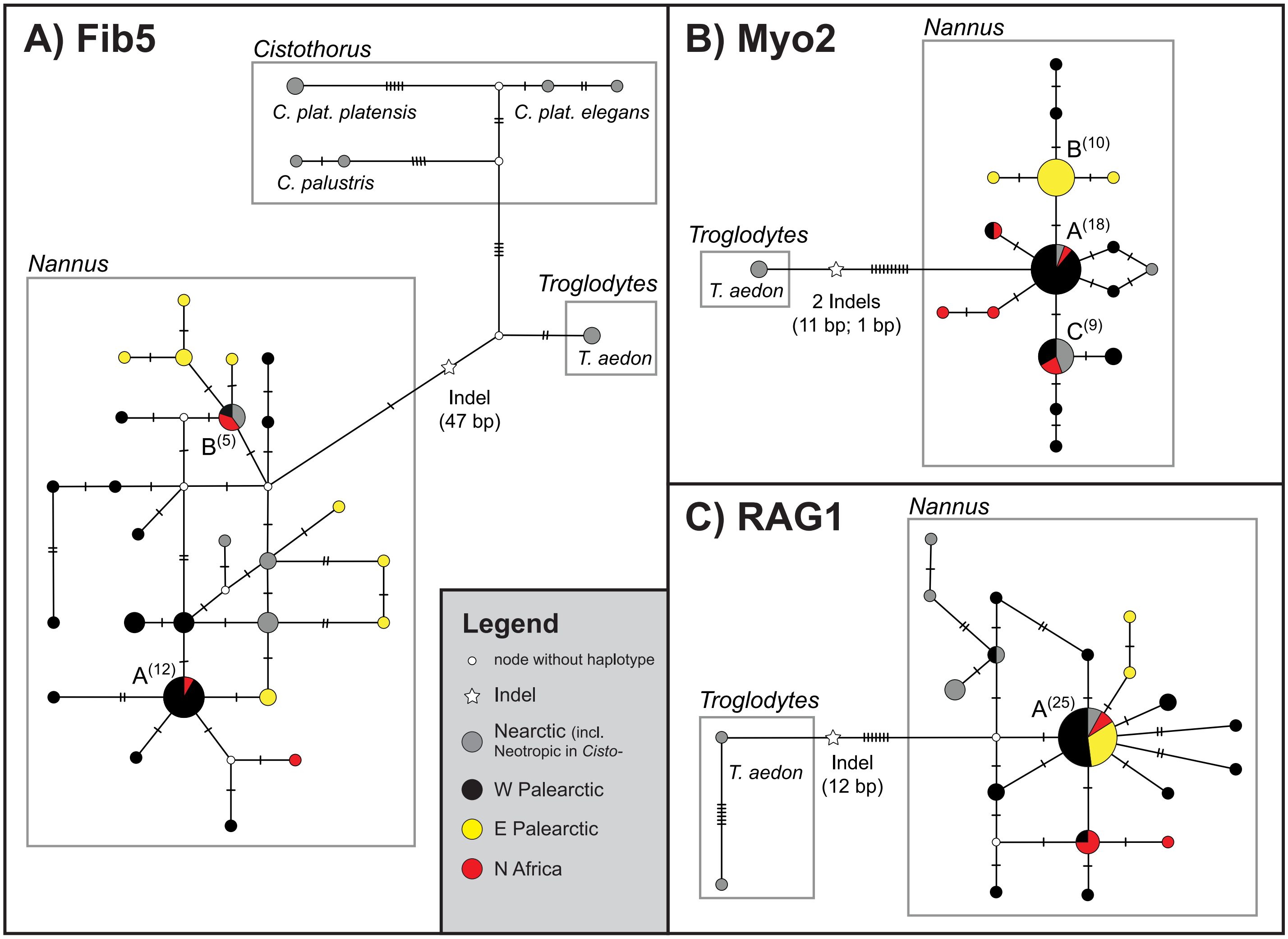

Fig 4. Allelic haplotype networks for three nuclear markers of *Nannus* spp. samples with *troglodytes aedon* as outgroup. A: *Fib5*, including *Cistothorus palustris* and *C. platensis* as outgroups (n = 31); B: *Myo2* (n = 27); C: *RAG1* (n = 26). Circles scaled to sample size (stated in parentheses for named allelic haplotypes); substitution distances not to scale.

https://doi.org/10.1371/journal.pone.0230151.g004

Multi-locus phylogeny and divergence times

Our multi-locus phylogeny (Fig 5; see S2 Fig for all outgroup taxa included; S3 Fig for ML reconstruction) supported the monophyly of the *Nannus pacificus/hiemalis/troglodytes* species complex as the sister clade of *Troglodytes aedon* (1.00 PP). According to our time calibration, the differentiation of the *Nannus* species complex from its sister clade took place about 7.4 Ma ago in the late Miocene (late Tortonian).

According to the fossil time calibration of the multi-locus tree, the onset of diversification among the three *Nannus* wren species was dated to the early Pleistocene at about 2.0 Ma. Divergence times inferred from mtDNA substitution rates suggested two basically different scenarios. The maximum uncorrected mean rate for *ND2* was in good accordance with an early Pleistocene onset of the *Nannus* radiation (2.0–2.5 Ma) inferred from the fossil calibration (Fig 2B). Partitioning of mtDNA genes by codon position had little if any effect on tmrca estimates. However, the effect of body mass correction was considerable because divergence time estimates inferred from mass-corrected rates were almost twice as old as those inferred

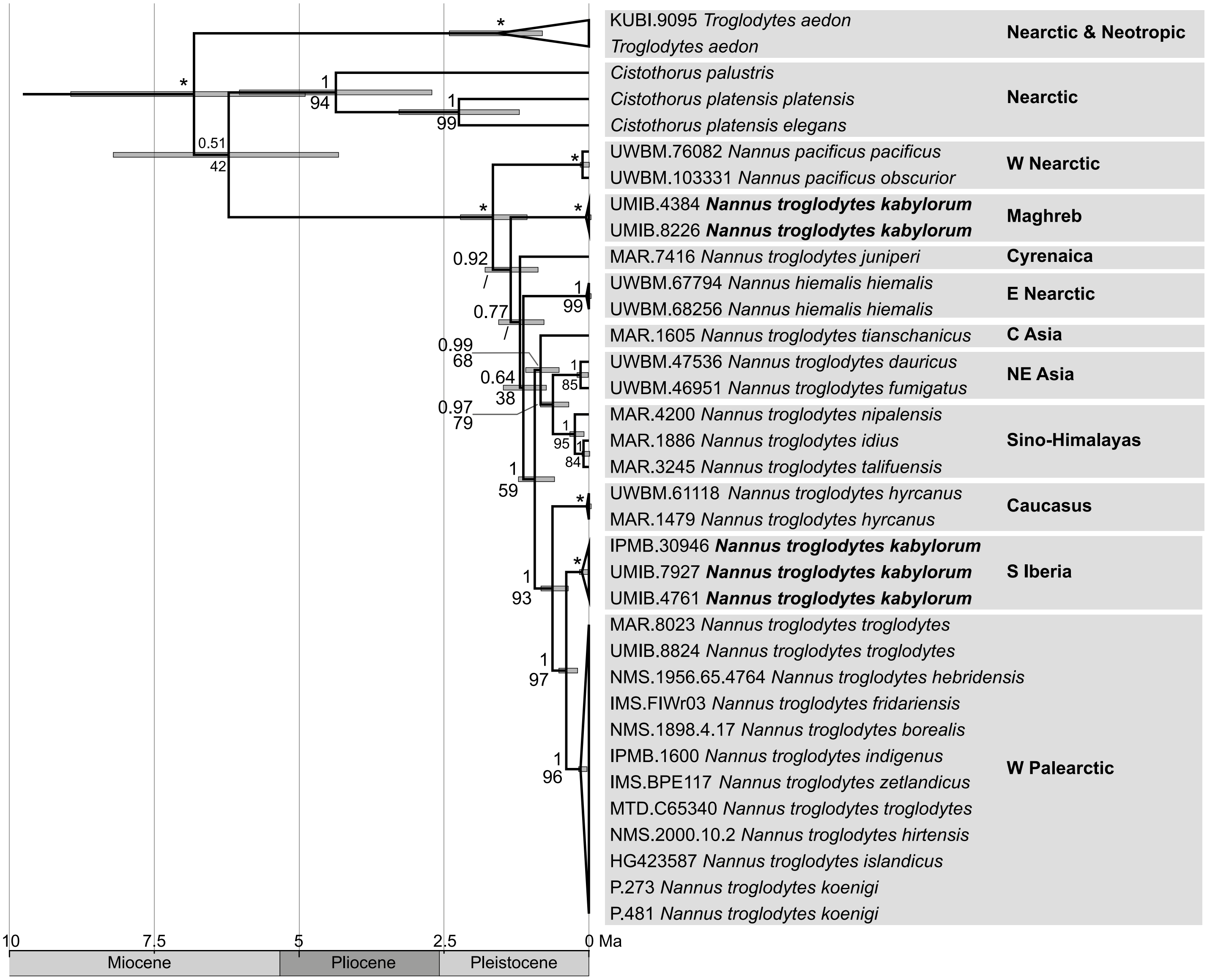

Fig 5. Multi-locus phylogeny of the *Nannus pacificus/hiemalis/troglodytes* species complex with outgroups reduced to *troglodytes aedon* and *Cistothorus* spp. (n = 35). Bayesian reconstruction across the five loci *COI* (696 bp), *ND2* (1033 bp), *Myo2* (736 bp), *Fib5* (570 bp), and *RAG1* (763 bp). Node support values indicate Bayesian posterior probabilities (above nodes) and bootstrap values of maximum likelihood analysis (below nodes); / = node was not recovered in the ML tree); asterisks mark nodes with full support from both analyses. Divergence times estimated with fossil calibration; 95% confidence intervals shown as node bars. Holocene (0.012–0 Ma, [81]) omitted on timescale for readability.

https://doi.org/10.1371/journal.pone.0230151.g005

from the minimum uncorrected rate and from fossil calibration (Fig 2B). According to mass-corrected rates, the basal radiation of *Nannus* started already in the early Pliocene (~ 5.0 Ma). Tmrca estimates inferred from the minimum uncorrected rate supported the Pliocene diversification of *Nannus*. That scenario was consistent for nearly all runs with mass-corrected rates for the entire *ND2* gene and for the 3rd codon position only (Fig 2B). Application of mass-corrected rates to the reduced Troglodytidae data set (all other families of Certhioidea and further outgroups excluded) yielded slightly younger divergence time estimates. This effect was strongest for mass-corrected rates for the 3rd codon position that yielded a tmrca estimate for *Nannus* wrens at the Pliocene-Pleistocene boundary (Fig 2B).

In the Bayesian tree (Fig 5), the two Nearctic *Nannus* species (*N. hiemalis*, *N. pacificus*) and the two North African clades (*N. t. kabylorum* from the Western Maghreb and *N. t. juniperi* from the Cyrenaica) appeared as four deeply split successively basal offshoots of the *Nannus* clade. Neither the Nearctic species nor the two North African clades appeared as sister clades. However, none of the basal nodes received strong support, so the phylogenetic relationships of the four taxa in question were not fully resolved. In the ML tree *N. t. juniperi* from the Cyrenaica appeared as a basal offshoot and the two successively basal splits were i) a clade uniting Maghrebian *N. t. kabylorum* and Nearctic *N. pacificus* with poor support for this sister group relationship, and ii) Nearctic *N. hiemalis* (S3 Fig).

The terminal clade comprising all samples of continental Eurasian *N. troglodytes* received full support in both BI and ML analysis (Figs 5 and S3). That Eurasian clade was divided in an Eastern and a Western Palearctic subclade that were both strongly supported. East-West divergence among the two Eurasian continental subspecies groups was dated to Pleistocene in all time calibrations (e.g. to about 0.9 Ma based on the fossil-calibration, to 1.2–1.3 Ma based on uncorrected *ND2* rate, and to slightly earlier periods at the Pliocene-Pleistocene boundary (2.8 Ma) based on mass-corrected rates. Each of the two Eurasian subclades comprised three major genetic lineages (Fig 5). In the Western Palearctic these are i) *N. t. hyrcanus* from the Caucasus that was sister to a terminal sister group of ii) *N. t. kabylorum* from southern Iberia and iii) the large nominate clade from continental Europe (nominate *N. t. troglodytes*), the British Isles (sspp. *zetlandicus*, *fridariensis*, *hebridensis*, *hirtensis*, *indigenus*), other Atlantic Islands (sspp. *islandicus*, *borealis*) and Corsica-Sardinia (ssp. *koenigi*). In the Eastern Palearctic three distinct clades are represented by i) *N. t. tianschanicus* from Central Asia that was sister to a terminal sister group of ii) populations from the Sino-Himalayas (sspp. *nipalensis*, *talifuensis*, *idius*) and iii) populations from the Russian Far East and Japan (sspp. *dauricus*, *fumigatus*).

Species distribution model for Western Palearctic *Nannus troglodytes*

The SDM for the current distribution of *N. troglodytes* in the Western Palearctic as well as the reconstructed SDMs for distributions during the LGM are shown in Fig 6. Model performance across all replicate runs was high for current (AUC test = 0.842) as well as past climatic conditions (CCSM3 = 0.838; MPI-ESM-P = 0.83; MIROC = 0.841) indicating that the model discriminates well between suitable and unsuitable space. Across all models, temperature seasonality (bio 4) had the highest contribution (31.4–34.1%), followed by precipitation of the driest quarter (bio 17, 21.4–21.4%), precipitation seasonality (bio 15, 17.7%), mean diurnal temperature range (bio 2, 8.2%), and maximum temperature of the warmest month (bio 5, 7.6%). Contribution of the remaining predictors did not exceed 5%. For details on variable contributions of all models see Table 1.

A comparison of the SDM for current and LGM conditions reveals that the extent of suitable space was considerably smaller during the LGM (Fig 6). Areas of high suitability indicate that the species range in the Western Palearctic could have contracted to isolated refugia situated in the Mediterranean region. A larger central refuge (or chain of smaller refuges) existed on the Italian Peninsula, the Balkan Peninsula, and some islands in the South of the continent (Corsica, Sardinia, Sicily, and Crete; Fig 6B–6D). Potential Iberian refugia could have existed rather in the West of the peninsula, whereas in the Eastern Mediterranean, small glacial refugia may have existed in the Levant and on Cyprus (Fig 6B–6D). In North Africa scattered glacial refugia might have existed in the Maghreb and these were largely separated from a small isolated refuge in the Cyrenaica according to all three models (Fig 6B–6D).

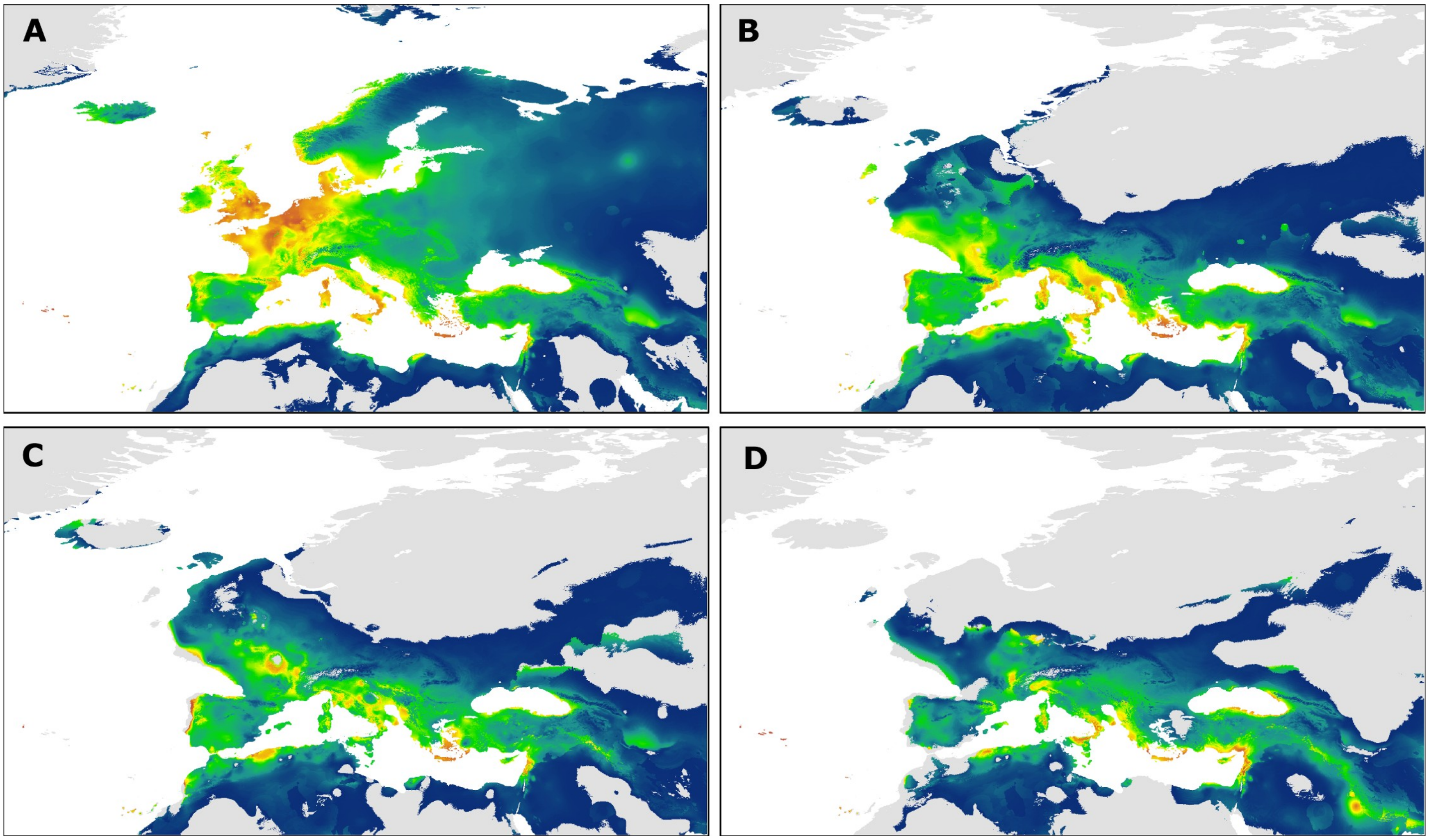

Fig 6. Results of species distribution modelling for Western Palearctic *Nannus troglodytes*. Current potential distribution as derived from Maxent (A). Projection onto climatic conditions of the Last Glacial Maximum as derived from the global circulation models MPI-ESM-P (B), CCSM3 (C), and MIROC-ESM (D). Suitability ranges from moderate (dark blue) to high (red).

https://doi.org/10.1371/journal.pone.0230151.g006

Discussion

According to our phylogenetic analysis, genetic diversification of *Nannus troglodytes* is considerably greater than previously documented. In addition to four known mitochondrial lineages on the Eurasian continent (Europe, Caucasus, Nepal, and Eastern Asia [35]) we identified further four distinct clades of the *N. troglodytes* phylogeny: two in the Ibero-Maghrebian region

Table 1. Contribution of selected environmental predictor variables. Variable contributions exceeding 5% are displayed in bold; temperature abbreviated as T.

Predictor	Predictor name	Variable contribution (%)			
		current	CCSM3	MPI-ESM-P	MIROC
**Bio 2**	Mean diurnal T range	**8.2**	**8.9**	**9.9**	**7.5**
**Bio 3**	Isothermality	3.4	3.8	3.6	4
**Bio 4**	T seasonality	**34.1**	**32.9**	**31.8**	**32.2**
**Bio 5**	Max. T of the warmest month	**7.6**	**9.0**	**9.6**	**8.5**
**Bio 8**	Mean T of the wettest quarter	2.7	2.8	2.4	3.5
**Bio 12**	Annual precipitation	4.8	3.8	4.1	4.9
**Bio 15**	Precipitation seasonality	**17.8**	**16.5**	**13.7**	**17.3**
**Bio 17**	Precipitation of the driest quarter	**21.4**	**22.3**	**24.8**	**22.1**
**Total**		**100**	**100**	**100**	**100**

https://doi.org/10.1371/journal.pone.0230151.t001

(two clades of *N. t. kabylorum*), one in the Cyrenaica (*N. t. juniperi*), and one in Central Asia (*N. t. tianschanicus*). Monophyly of *N. troglodytes* was not supported by our multi-locus analysis and received only poor support from the single-locus analysis by Drovetski *et al.* ([35]; who excluded the two North African clades that rendered *N. troglodytes* paraphyletic). The two Nearctic species *N. pacificus* and *N. hiemalis* did not result as sister clades either (this study; [35]); yet there are also examples for non-monophyletic Nearctic species groups from other Holarctic passerine genera, e.g. kinglets, *Regulus* [117], and nuthatches, *Sitta* [118]. However, in wrens, none of the three basal nodes of the *Nannus* clade received reasonable support. Therefore, no firm conclusions on colonization pathways between the Nearctic and the Palearctic can be drawn from this poorly supported topology. Drovetski *et al.* [35] postulated five Pleistocene vicariant events that triggered diversification of *Nannus* wrens into six clades from a single Holarctic ancestor. In accordance with Drovetski *et al.* [35], our divergence time estimates inferred from fossil dating suggested that *Nannus* wrens started diversifying in the early Pleistocene when Nearctic, Eurasian and North African lineages separated from each other during a very short time period (2.0–1.2 Ma). We advocate for the Pleistocene scenario of *Nannus* wren diversification as the more reliable one, because it is supported by i) tmrca estimates based on the maximum uncorrected *ND2* rate for passerine birds [94] whereas the minimum uncorrected *ND2* rate was based on a mixed data set of Passeriformes and non-Passeriformes [96,119] and would thus be expected to pre-date passerine lineage splits to implausibly older ages; ii) tmrca estimates based on the mass-corrected *ND2* rate for the *Nannus* data set only (molecular clock calibration based on mass-corrected rates should rely on data sets of closely related species of comparable body mass [99]).

Though most tmrca estimates based on mass-corrected substitution rates for *ND2* suggested a more ancient (Pliocene) basal split of *Nannus*, early diversification on the Eurasian continent (basal split of the terminal crown clade) was unanimously dated to the Pleistocene in all our calibrations (see below). In the Eastern Palearctic, three major clades diversified during the Pleistocene in a circum-Tibetan phylogeographic pattern, that has been documented for many other passerine birds [120–122]: i) a Central Asian clade, ii) a Far East Russian/Japanese clade, and iii) a Sino-Himalayan clade. In the latter, the split between *N. t. nipalensis* from Nepal vs. Chinese *N. t. idius* and *N. t. talifuensis* corresponds to a characteristic east-west disjunction found in many other birds as well [120,121,123].

In the Western Palearctic however, the most striking diversification patterns were observed in the Ibero-Maghrebian and Cyrenaican regions.

Phylogeographic patterns in the Ibero-Maghrebian and Cyrenaican regions

We could identify strongly divergent mtDNA lineages of Cyrenaican, Maghrebian, and Iberian wren populations. In our phylogeny, the two North African clades (*N. t. kabylorum* in the Maghreb and *N. t. juniperi* in the Cyrenaica) appeared as two early and deep splits from the *N. troglodytes* clade; however their phylogenetic relationships remained unresolved.

Biogeographical affinities between the Iberian Peninsula and North Africa are characterized by complex phylogeographical patterns and multiple phases of trans-Mediterranean bidirectional faunal exchange during Pliocene and Pleistocene times [12]. Strong genetic differentiation of Northwest African populations from closest Eurasian relatives as found in the Eurasian Wren is a common phylogeographic pattern in several other Palearctic bird taxa, such as *Strix aluco* [124–126], *Picus* spp. [127], *Pica* spp. [128], *Periparus ater* [129], *Cyanistes* spp. [27–29], *Acrocephalus scirpaceus* [130,131], *Certhia brachydactyla* [132], *Cinclus cinclus* [133], *Muscicapa striata* [134], *Ficedula* spp. [135,136], *Fringilla coelebs* [137,138], and *Loxia* spp. [139]. Similarly deep splits between Iberian and Maghrebian populations of the same species were

found in other terrestrial vertebrates, such as in amphibians, reptiles [25,140], and in mammals [141–144]. The Strait of Gibraltar was suggested to be an effective biogeographic barrier for trans-Mediterranean floral and faunal interchange [145–147] and accordingly split ages between populations north and south of the Strait were often dated to times when land bridges between the two continents existed, e.g. during the Messinian Salinity Crisis (MSC) [23,148]. Earlier divergence times for the basal splits of Ibero-Maghrebian populations (*N. t. kabylorum* and *N. t. juniperi*) inferred from mass-corrected rates would be in accordance with that Pliocene scenario. There is even strong evidence for earlier Ibero-Maghrebian interchange prior to the MSC, e.g. in mammals [149], but also for more recent trans-Mediterranean faunal exchange including oversea-dispersal, stepping-stone colonization during phases of low sea-level [150,151] or even through human-mediated dispersal [152].

However, the entire Eurasian *Nannus* wren radiation was dated to the Pliocene-Pleistocene boundary and therefore the remarkable divergence between and within the East and West Palearctic clades was presumably shaped by range fragmentation and range shifts along with global climate cooling. Accordingly, phylogeographic patterns on the Eurasian continent, in the Mediterranean Basin, and in North Africa coincide with major glacial refugia as identified in our SDM analysis (i.e. the southern European peninsulas [4,153]).

In North Africa, Pleistocene origin of an east-west disjunction as suggested for *N. troglodytes* was suggested for several other vertebrate species, such as in reptiles (e.g. in chelonians: *Testudo graeca* [154]; and in lizards: *Acanthodactylus pardalis* group [155]), in birds (e.g. *Cyanistes teneriffae* [27–29], *Galerida cristata* [156], and *Fringilla coelebs* [30]) and in mammals (e.g. *Jaculus orientalis* [157]). Rather complex phylogeographic patterns have been documented for other mammals, e.g. vicariance of genetically distinct Red Fox populations (*Vulpes vulpes*) of the Maghreb and the Fertile Crescent that are replaced by Rueppell's Fox (*Vulpes rueppellii*) across their distribution gap in Libya [143]. In other mammal species, Sardinian and other Mediterranean island populations are firmly nested in a trans-Maghrebian clade (e.g. in the European Wildcat, *Felis silvestris* [144]), or in a distinctive East Maghrebian clade (e.g. in the Greater White-toothed Shrew, *Crocidura russula* [158]).

Even if we considered an earlier onset of *Nannus* radiation (as inferred from mass-corrected *ND2* rates) we can assume that North African wren populations were affected by environmental changes along with the global cooling towards the end of the Pliocene and during the Pleistocene, e.g. during phases of aridification in North Africa at 2.8 Ma and 1.7 Ma [159,160]. As the establishment of Pleistocene glacial/interglacial climatic cycles resulted in the alternation of humid- and arid-adapted vegetation in the Mediterranean Basin [159–161], wrens might have occupied a broader range along the North African coastline during a relatively humid phase providing suitable bridging habitats and then have retreated into the Maghrebian Atlas Mountains and the Cyrenaican Jebel Akhdar massif due to the onset of increasing lowland aridification. Paleoenvironmental studies suggested that during the late Pleistocene the Jebel Akhdar constituted an "environmental refugium" from the extreme arid conditions in the neighbouring Sahara [162–164]. According to all three models compared in our SDM analysis, suitable habitats for *N. troglodytes* in the Maghreb and in the Cyrenaica were separated by a large corridor of unsuitable habitat (clearest separation according to CCSM3 and MIROC-ESM; Fig 6C and 6D). Thus, the extant distribution of *N. troglodytes* in North Africa well corresponds to a postulated Mauritanian and a Cyrenaican refugium [12].

Cryptic lineages in Western Palearctic wrens

The remaining *N. troglodytes* taxa of the Eurasian continent showed a typical differentiation into Western and Eastern Palearctic sister clades, which is a common differentiation pattern of

Palearctic birds observed on different taxonomic levels, such as within species (e.g. in tits [165] and in magpies [128]) or between closely related species pairs (e.g. in corvids [166] and in buntings [167]).

Within the Western Palearctic clade of Eurasian Wrens, we identified the Caucasian clade of *N. t. hyrcanus* as a first basal offshoot. According to DNA barcoding, there is first evidence that *N. t. cypriotes* of the eastern Mediterranean (distributed on Aegean Islands, western and southern Anatolia, Cyprus, and Levantine coastline [38]), is closely related to the Caucasian lineage (S1 Fig) [168]. However, the existence of another distinct mtDNA lineage (of *N. t. cypriotes*) in the eastern Mediterranean Basin needs further support from future analyses based on a broader sampling. Further potential glacial refugia in the eastern Mediterranean and the Middle East identified by our SDM analyses could have harboured ancestors of extant *N. t. cypriotes* and *N. t. hyrcanus* (Fig 6).

In a terminal late Pleistocene colonization event, founder populations from the European continent spread to the North Atlantic islands [49,50]. According to our SDM analyses suitable glacial refugia in south-western Europe could have harboured founder populations that colonized the British Islands via land bridges during the LGM (Fig 6B–6D).

On the Iberian Peninsula, we found a north-south disjunction between southern Iberian populations of *N. t. kabylorum* that represent a cryptic phylogenetic lineage and northern populations of the nominate ssp. *troglodytes* that were nested in the European nominate clade. Strong genetic divergence of two mtDNA lineages on the Iberian Peninsula was also observed in many plant and animal species (reviewed in [4,10]), including e.g. amphibians [140,169], mammals [170], and bird species such as the White-throated Dipper, *Cinclus cinclus* [133,171,172] and Savi's Warbler, *Locustella luscinoides* [173]. Particularly, in the latter species local admixture of two divergent mtDNA lineages in Iberian population is striking; however, due to low sample sizes on the Iberian Peninsula in our study we cannot infer reliable information on levels of mitochondrial introgression. This must be subject to future population genetic analyses based on a comprehensive sampling of Iberian populations.

The Iberian Peninsula is regarded as one major Pleistocene glacial refugium in the Western Palearctic and as an important origin of post-glacial expansion of species to Central Europe. According to our SDM analysis isolated areas of suitable habitat during the LGM existed only along the west coast of the Iberian Peninsula. Ancestors of the extant genetically distinct *N. t. kabylorum* populations from southern Spain might have survived the Pleistocene in that region.

Along the southern European coastline a long chain of suitable habitat for *N. troglodytes* has existed during the LGM (Fig 6B–6D), e.g. in two other classical European glacial refugia (on the Apennine Peninsula and on the Balkan Peninsula). These central Mediterranean refugia must have been occupied by ancestral populations of the nominate *troglodytes* clade who later re-colonized large parts of Europe (including the Atlantic islands and the northern parts of the Iberian Peninsula) in one post-glacial expansion event. Very likely, Holocene range expansion of *N. t. troglodytes* to northern Iberia has limited the northward dispersal of the Iberian relict populations (i.e. the cryptic ssp. *kabylorum* lineage from southern Iberia) and shaped the intra-Iberian differentiation we observe today.

A similar pattern of post-glacial range expansion has been inferred for the Dunnock, *Prunella modularis* [174]. In this species, northern Europe appears to have been colonised from Apennine and Balkan refugia, rather than from genetically distinct Iberian or Caucasian lineages. Considering the heterogeneous distribution of wrens in Iberia with a continuous distribution in the north and rather scattered distribution in the southern part [175,176], this distributional pattern might already reflect genetic differentiation of wren lineages. Whether a zone of sympatry exists between the ranges of the cryptic south Iberian "*kabylorum*"-

populations and north Iberian *troglodytes*-populations, is not known to date and would be subject to more detailed future research.

Supporting information

S1 Fig. TCS haplotype network drawn for the mitochondrial barcoding marker *COI* (551 bp) of the *Nannus pacificus/hiemalis/troglodytes* species complex. For n = 81 sequences, including 18 subspecies of *N. troglodytes*. Haplotype circles scaled to sample size (n) of each represented haplotype; substitution distances not to scale.
(JPG)

S2 Fig. Multi-locus phylogeny of the *Nannus pacificus/hiemalis/troglodytes* species complex with all outgroups included. Bayesian reconstruction across the five loci *COI*, *ND2*, *Myo2*, *Fib5*, and *RAG1*. Node support values indicate Bayesian posterior probabilities. Divergence times estimated with fossil calibration. 95% confidence intervals shown as node bars. Holocene (0.012–0 Ma, [87]) omitted on timescale for readability.
(JPG)

S3 Fig. Maximum likelihood phylogeny as calculated with RAxML v.7.2.6. Node values indicate bootstrap support.
(JPG)

S1 Table. Overview of newly sequenced samples of the *Nannus troglodytes/hiemalis/pacificus* species group and further outgroup taxa used for this study. GenBank accession numbers are given for each of the used markers (XXX = sequence data from own samples; accession numbers will be provided upon manuscript acceptance). Highlited samples (orange) were included in the multi-locus phylogenetic reconstruction; further samples (imported from GenBank) included in haplotype networks (*ND2* & *COI*) only. Geographic coordinates are given in parentheses if estimated. Abbreviations of collections and institutions (Inst.) for newly sequenced samples as follows (in alphabetical order): Burke Museum of Natural History and Culture (UWBM), University of Washington, Seattle, WA, USA; Institute of Medical Sciences (IMS), University of Aberdeen, Foresterhill, Aberdeen, UK; Institute of Pharmacy and Molecular Biotechnology (IPMB), Heidelberg University, Heidelberg, Germany; Muséum National d'Histoire Naturelle (MNHN), Paris, France; National Museums Scotland (NMS), Edinburgh, UK; Research Unit of Biodiversity (UMIB), Oviedo University, Mieres, Spain; Senckenberg Natural History Collections Dresden (SNSD), Museum of Zoology, Dresden, Germany; tissue collection of J. Martens (MAR).
(XLSX)

S2 Table. Summary of primer pairs used for PCR and sequencing, together with respective PCR thermal cycle settings.
(XLSX)

Acknowledgments

We are indebted to S. Birks (Burke Museum of Natural History and Culture, Seattle, WA, USA) and to J.-M. Pons (L'Institut de Systématique, Évolution, Biodiversité, Muséum national d'histoire naturelle, Paris, France), who kindly provided access to genetic material from their collections. We also thank G. López and CHAGRA ringing group for providing samples.

All genetic analyses were performed at SGN-SNSD-Mol-Lab and we are grateful to A. Rauh, C. Spitzweg, A. Müller, and C. Kehlmaier for practical help in the molecular lab facilities. We would also like to thank M. Vamberger for support in phylogenetic analyses.

Finally, we would like to thank four anonymous reviewers for constructive criticism on the manuscript.

Author Contributions

Conceptualization: Jens Hering, Martin Päckert.

Data curation: Frederik Albrecht, Martin Päckert.

Formal analysis: Frederik Albrecht, Juan Carlos Illera, Flora Ihlow, Martin Päckert.

Funding acquisition: Juan Carlos Illera, Jochen Martens, Martin Päckert.

Investigation: Frederik Albrecht, Thomas J. Shannon.

Methodology: Flora Ihlow.

Project administration: Frederik Albrecht, Martin Päckert.

Resources: Jens Hering, Elmar Fuchs, Juan Carlos Illera, Thomas J. Shannon, J. Martin Collinson, Michael Wink, Jochen Martens, Martin Päckert.

Supervision: Martin Päckert.

Validation: Martin Päckert.

Visualization: Frederik Albrecht, Flora Ihlow.

Writing – original draft: Frederik Albrecht.

Writing – review & editing: Frederik Albrecht, Jens Hering, Elmar Fuchs, Juan Carlos Illera, Flora Ihlow, Thomas J. Shannon, J. Martin Collinson, Michael Wink, Jochen Martens, Martin Päckert.

References

1. Myers N, Mittermeier RA, Mittermeier CG, Da Fonseca GA, Kent J. Biodiversity hotspots for conservation priorities. Nature. 2000; 403: 853–858. https://doi.org/10.1038/35002501 PMID: 10706275
2. Cuttelod A, García N, Abdul Malak D, Temple H, Katariya V. The Mediterranean: a biodiversity hotspot under threat. In: Vié J-C, Hilton-Taylor C, Stuart SN, editors. The 2008 Review of The IUCN Red List of Threatened Species. IUCN Gland; 2008. pp. 89–101.
3. Blondel J, Aronson J, Bodiou JY, Boeuf G. The Mediterranean Region: Biological Diversity in Space and Time. 2nd ed. Oxford: Oxford University Press; 2010.
4. Hewitt GM. Mediterranean Peninsulas: The Evolution of Hotspots. In: Zachos FE, Habel JC, editors. Biodiversity Hotspots. Berlin: Springer; 2011. pp. 123–147. https://doi.org/10.1007/978-3-642-20992-5_7
5. Marchese C. Biodiversity hotspots: A shortcut for a more complicated concept. Glob Ecol Conserv. 2015; 3: 297–309. https://doi.org/10.1016/j.gecco.2014.12.008
6. Ducci F, Cutino I, Monteverdi MC, Picard N, Proietti R. Marginal/peripheral populations of forest tree species and their conservation status: report for Mediterranean region. Ann Silvicult Res. 2017; 41: 31–40. https://doi.org/10.12899/asr-1533
7. Eliades NGH, Aravanopoulos FA, Christou AK. Mediterranean Islands Hosting Marginal and Peripheral Forest Tree Populations: The Case of *Pinus brutia* Ten. in Cyprus. Forests. 2018; 9: 514. https://doi.org/10.3390/f9090514
8. Medail F, Quézel P. Hot-spots analysis for conservation of plant biodiversity in the Mediterranean Basin. Ann Mo Bot Gard. 1997; 84: 112–127. https://doi.org/10.2307/2399957
9. Médail F, Quézel P. Biodiversity hotspots in the Mediterranean Basin: setting global conservation priorities. Conserv Biol. 1999, 13: 1510–1513. https://doi.org/10.1046/j.1523-1739.1999.98467.x
10. Gómez A, Lunt DH. Refugia within refugia: patterns of phylogeographic concordance in the Iberian Peninsula. In: Weiss S, Ferrand N, editors. Phylogeography of Southern European Refugia. Dordrecht: Springer; 2007. pp. 155–188.

11. Médail F, Diadema K. Glacial refugia influence plant diversity patterns in the Mediterranean Basin. J Biogeogr. 2009; 36: 1333–1345. https://doi.org/10.1111/j.1365-2699.2008.02051.x
12. Husemann M, Schmitt T, Zachos FE, Ulrich W, Habel JC. Palaearctic biogeography revisited: evidence for the existence of a North African refugium for Western Palaearctic biota. J Biogeogr. 2014; 41: 81–94. https://doi.org/10.1111/jbi.12180
13. Phoca-Cosmetatou N, Rabett RJ. Pleistocene island occupation in the Mediterranean: Insights from a tied-biome approach to glacial refugia. In: Boyle K, Rabett RJ, Hunt CO, editors. Living in the landscape: Essays in honour of Graeme Barker. McDonald Institute Monographs. Cambridge: McDonald Institute for Archaeological Research; 2014. pp. 83–108.
14. Dobson M, Wright A. Faunal relationships and zoogeographical affinities of mammals in north-west Africa. J Biogeogr. 2000; 27: 417–424. https://doi.org/10.1046/j.1365-2699.2000.00384.x
15. Beddek M, Zenboudji-Beddek S, Geniez P, Fathalla R, Sourouille P, Arnal V, et al. Comparative phylogeography of amphibians and reptiles in Algeria suggests common causes for the east-west phylogeographic breaks in the Maghreb. PloS One. 2018; 13: e0201218. https://doi.org/10.1371/journal.pone.0201218 PMID: 30157236
16. Buforn E, De Galdeano CS, Udías A. Seismotectonics of the Ibero-Maghrebian region. Tectonophysics. 1995; 248: 247–261. https://doi.org/10.1016/0040-1951(94)00276-F
17. Buforn E, Udías A, Pro C. Large earthquakes at the Ibero-Maghrebian region: Basis for an EEWS. Pure Appl Geophys. 2015; 172: 2387–2396. https://doi.org/10.1007/s00024-014-0954-0
18. Negredo AM, Bird P, Sanz de Galdeano C, Buforn E. Neotectonic modeling of the Ibero-Maghrebian region. J Geophys Res: Solid Earth. 2002; 107: B11. https://doi.org/10.1029/2001JB000743
19. Faille A, Andújar C, Fadrique F, Ribera I. Late Miocene origin of an Ibero-Maghrebian clade of ground beetles with multiple colonizations of the subterranean environment. J Biogeogr. 2014; 41: 1979–1990. https://doi.org/10.1111/jbi.12349
20. Escoriza D, Hassine JB, Sala J, Boix D. Zoophagy in the larvae of Ibero-Maghrebian spade-foot toads. Herpetologica. 2016; 72: 281–287. https://doi.org/10.1655/HERPETOLOGICA-D-16-00001.1
21. Pokrant F, Kindler C, Ivanov M, Cheylan M, Geniez P, Böhme W, et al. Integrative taxonomy provides evidence for the species status of the Ibero-Maghrebian grass snake *Natrix astreptophora*. Biol J Linn Soc. 2016; 118: 873–888. https://doi.org/10.1111/bij.12782
22. Gutiérrez-Rodríguez J, Barbosa AM, Martínez-Solano Í. Integrative inference of population history in the Ibero-Maghrebian endemic *Pleurodeles waltl* (Salamandridae). Mol Phylogenetics Evol. 2017; 112: 122–137. https://doi.org/10.1016/j.ympev.2017.04.022 PMID: 28454930
23. Kindler C, de Pous P, Carranza S, Beddek M, Geniez P, Fritz U. Phylogeography of the Ibero-Maghrebian red-eyed grass snake (*Natrix astreptophora*). Org Divers Evol. 2018; 18: 143–150. https://doi.org/10.1007/s13127-017-0354-2
24. Berrahmouni N, Regato P. WWF Ecoregions: Northern Africa: Algeria and Morocco and Tunisia. Available from: https://www.worldwildlife.org/ecoregions/pa0513. Cited 01 February 2020.
25. Stuckas H, Velo-Antón G, Fahd S, Kalboussi M, Rouag R, Arculeo M, et al. Where are you from, stranger? The enigmatic biogeography of North African pond turtles (*Emys orbicularis*). Org Divers Evol. 2014; 14: 295–306. https://doi.org/10.1007/s13127-014-0168-4
26. Nicolas V, Mataame A, Crochet PA, Geniez P, Ohler A. Phylogeographic patterns in north African water frog *Pelophylax saharicus* (Anura: Ranidae). J Zool Syst Evol Res. 2015; 53: 239–248. https://doi.org/10.1111/jzs.12094
27. Päckert M, Martens J, Hering J, Kvist L, Illera JC. Return flight to the Canary Islands–the key role of peripheral populations of Afrocanarian blue tits (Aves: *Cyanistes teneriffae*) in multi-gene reconstructions of colonization pathways. Mol Phylogenetics Evol. 2013; 67: 458–467. https://doi.org/10.1016/j.ympev.2013.02.010 PMID: 23454090
28. Gohli J, Leder EH, Garcia-del-Rey E, Johannessen LE, Johnsen A, Laskemoen T, et al. The evolutionary history of Afrocanarian blue tits inferred from genomewide SNPs. Mol Ecol. 2015; 24: 180–191. https://doi.org/10.1111/mec.13008 PMID: 25407440
29. Stervander M, Illera JC, Kvist L, Barbosa P, Keehnen NP, Pruisscher P et al. Disentangling the complex evolutionary history of the Western Palearctic blue tits (*Cyanistes* spp.)–phylogenomic analyses suggest radiation by multiple colonization events and subsequent isolation. Mol Ecol. 2015; 24: 2477–2494. https://doi.org/10.1111/mec.13145 PMID: 25753616
30. Perktaş U, Peterson AT, Dyer D. Integrating morphology, phylogeography, and ecological niche modeling to explore population differentiation in North African Common Chaffinches. J Ornithol. 2017; 158: 1–13. https://doi.org/10.1007/s10336-016-1361-3
31. Wrens Brewer D., Dippers and Thrashers. London: Christopher Helm; 2001.

32. Kroodsma DE, Brewer D. Northern Wren *Troglodytes troglodytes*. In: del Hoyo J, Elliot A, Christie DA, editors. Handbook of the Birds of the World, vol. 10, Cuckoo-shrikes to Thrushes. Barcelona: Lynx Edicions; 2005. pp. 432–433.
33. del Hoyo J, Collar NJ, Christie DA, Elliott A, Fishpool LDC, Boesman P, et al., editors. HBW and BirdLife International Illustrated Checklist of the Birds of the World, vol. 2, Passerines. Barcelona: Lynx Edicions; 2005.
34. Dallmann M. Der Zaunkönig: *Troglodytes troglodytes*. Die Neue Brehm-Bücherei 577. Wittenberg Lutherstadt: Ziemsen; 1987.
35. Drovetski SV, Zink RM, Rohwer S, Fadeev IV, Nesterov EV, Karagodin I, et al. Complex biogeographic history of a Holarctic passerine. Proc R Soc Lond B, Biol. Sci. 2004; 271: 545–551. https://doi.org/10.1098/rspb.2003.2638 PMID: 15129966
36. Kroodsma DE. Winter Wren singing behavior: A pinnacle of song complexity. Condor. 1980; 82: 357–365. https://doi.org/10.2307/1367556
37. Chesser RT, Banks RC, Barker FK, Cicero C, Dunn JL, Kratter AW, et al. Fifty-First Supplement to the American Ornithologists' Union Check-List of North American Birds. Auk. 2010; 127: 726–744. https://doi.org/10.1525/auk.2010.127.3.726
38. Dickinson EC, Christidis L, editors. The Howard and Moore Complete Checklist of the Birds of the World, 4th ed., vol. 2, Passerines. Eastbourne: Aves Press; 2014.
39. Gill F, Donsker D. IOC World Bird List (v 8.1). 2018. Available from: https://doi.org/10.14344/IOC.ML.8.1. Cited 20 February 2018.
40. Leukering T, Pieplow N. Pacific and Winter Wrens. Colorado Birds. 2010; 44: 281–286.
41. Sibley DA. Distinguishing Pacific and Winter Wrens. 2010. Available from: http://www.sibleyguides.com/2010/08/distinguishing-pacific-and-winter-wrens. Cited 01 October 2018.
42. Toews DP, Irwin DE. Cryptic speciation in a Holarctic passerine revealed by genetic and bioacoustic analyses. Mol Ecol. 2008; 17: 2691–2705. https://doi.org/10.1111/j.1365-294X.2008.03769.x PMID: 18444983
43. Barker FK. Molecular phylogenetics of the wrens and allies (Passeriformes: Certhioidea), with comments on the relationships of *Ferminia*. Am Mus Novit. 2017; 3887: 1–28. https://doi.org/10.1206/3887.1
44. Rice NH, Peterson AT, Escalona-Segura G. Phylogenetic patterns in montane *Troglodytes* wrens. Condor. 1999; 101: 446–451. https://doi.org/10.2307/1370013
45. Gómez JEM, Barber BR, Peterson AT. Phylogenetic position and generic placement of the Socorro Wren (*Thryomanes sissonii*). Auk. 2005; 122: 50–56. https://doi.org/10.1642/0004-8038(2005)122[0050:PPAGPO]2.0.CO;2
46. Baird SF. General report upon the zoology of the several Pacific railroad routes, part 2: Birds. Reports of explorations and surveys to ascertain the most practicable and economical route for a railroad from the Mississippi River to the Pacific Ocean. 1858;9: 367. https://doi.org/10.5962/bhl.title.11139
47. Clements JF, Schulenberg TS, Iliff MJ, Roberson D, Fredericks TA, Sullivan BL, et al. The eBird/Clements checklist of birds of the world: v2018. Available from: http://www.birds.cornell.edu/clementschecklist/download. Cited 18 February 2019.
48. Cramp S, editor. Handbook of the Birds of Europe, the Middle East and North Africa. The Birds of the Western Palearctic, vol. V. Tyrant Flycatchers to Thrushes. Oxford: Oxford University Press; 1988.
49. Shannon TJ, McGowan RY, Zonfrillo B, Piertney S, Collinson JM. A genetic screen of the island races of Wren *Troglodytes troglodytes* in the North-east Atlantic. Bird Study. 2014; 61: 135–142. https://doi.org/10.1080/00063657.2014.894493
50. Amouret J, Barisas DAG, Hallgrímsson GT, Summers RW, Pálsson S. Genetic divergence of *Troglodytes troglodytes islandicus* from other subspecies of Eurasian wren in northwestern Europe. J Avian Biol. 2016; 47: 26–35. https://doi.org/10.1111/jav.00744
51. Hartert E. On the Birds of Cyrenaica. Nov Zool. 1923; 30: 1–32.
52. Stanford JK. A survey of the ornithology of northern Libya. Ibis. 1954; 96: 449–473 & 606–624. https://doi.org/10.1111/j.1474-919X.1954.tb02336.x
53. Massa B. New and less known birds from Libya. Bull Br Ornithol Club. 1999; 119: 129–133. Available from: http://direct.biostor.org/reference/111958.
54. Isenmann P, Hering J, Brehme S, Essghaier M, Etayeb K, Bourass E, et al. Oiseaux de Libye/Birds of Libya. Paris: Société d'Etudes Ornithologiques de France (SEOF), Museum National d'Histoire Naturelle; 2016.

55. Hung CM, Drovetski SV, Zink RM. Matching loci surveyed to questions asked in phylogeography. Proc R Soc Lond B, Biol Sci. 2016; 283: 20152340. https://doi.org/10.1098/rspb.2015.2340 PMID: 26962145

56. BirdLife International & Handbook of the Birds of the World, 2016 [cited 17 June 2017]. *Troglodytes troglodytes* distributional area shapefile. The IUCN Red List of Threatened Species. Version 2016.1. Available from http://www.iucnredlist.org.

57. Kerr KC, Birks SM, Kalyakin MV, Red'kin YA, Koblik EA, Hebert PD. Filling the gap—COI barcode resolution in eastern Palearctic birds. Front Zool. 2009; 6: 29. https://doi.org/10.1186/1742-9994-6-29 PMID: 20003213

58. Tamura K, Stecher G, Peterson D, Filipski A, Kumar S. MEGA6: molecular evolutionary genetics analysis version 6.0. Mol Biol Evol. 2013; 30: 2725–2729. https://doi.org/10.1093/molbev/mst197 PMID: 24132122

59. Leigh JW, Bryant D. POPART: full-feature software for haplotype network construction. Methods Ecol Evol. 2015; 6: 1110–1116. https://doi.org/10.1111/2041-210X.12410

60. Rozas J, Ferrer-Mata A, Sánchez-DelBarrio JC, Guirao-Rico S, Librado P, Ramos-Onsins SE, et al. DnaSP 6: DNA sequence polymorphism analysis of large data sets. Mol Biol Evol. 2017; 34: 3299–3302. https://doi.org/10.1093/molbev/msx248 PMID: 29029172

61. Clement M, Posada DCKA, Crandall KA. TCS: a computer program to estimate gene genealogies. Mol Ecol. 2000; 9: 1657–1659. https://doi.org/10.1046/j.1365-294x.2000.01020.x PMID: 11050560

62. Clement M, Snell Q, Walker P, Posada D, Crandall K. TCS: Estimating gene genealogies. Parallel and Distributed Processing Symposium, International Proceedings. 2002; 2: 184. https://doi.org/10.1109/IPDPS.2002.1016585

63. Robbins MB, Nyári ÁS. Canada to Tierra del Fuego: species limits and historical biogeography of the Sedge Wren (*Cistothorus platensis*). Wilson J Ornithol. 2014; 126: 649–662. https://doi.org/10.1676/13-162.1

64. Lanfear R, Calcott B, Ho SY, Guindon S. PartitionFinder: combined selection of partitioning schemes and substitution models for phylogenetic analyses. Mol Biol Evol. 2012; 29: 1695–1701. https://doi.org/10.1093/molbev/mss020 PMID: 22319168

65. Drummond AJ, Rambaut A. BEAST: Bayesian evolutionary analysis by sampling trees. BMC Evol Biol. 2007; 7: 214. https://doi.org/10.1186/1471-2148-7-214 PMID: 17996036

66. Heled J, Drummond AJ. Calibrated Birth–Death Phylogenetic Time-Tree Priors for Bayesian Inference. Syst Biol. 2015; 64:369–383. https://doi.org/10.1093/sysbio/syu089 PMID: 25398445

67. Ritchie AM, Lo N, Ho SYW. The impact of tree prior on molecular dating of data sets containing a mixture of inter- and intraspecies sampling. Syst Biol. 2017; 66: 413–425. https://doi.org/10.1093/sysbio/syw095 PMID: 27798404

68. Sarver BAJ, Pennell MW, Brown JW, Keeble S, Hardwick KM, Sullivan J, et al. The choice of tree prior and molecular clock does not substantially affect phylogenetic inferences of diversification rates. PeerJ. 2019; 7: e6334. https://doi.org/10.7717/peerj.6334 PMID: 30886768

69. Turner AH, Pritchard AC, Matzke NJ. Empirical and Bayesian approaches to fossil-only divergence times: A study across the reptile clades. PLOS ONE. 2017; 12: e0169885. https://doi.org/10.1371/journal.pone.0169885 PMID: 28187191

70. Rambaut A, Drummond AJ, Xie D, Baele G, Suchard MA. Posterior summarisation in Bayesian phylogenetics using Tracer 1.7. Syst Biol. 2018: syy032. https://doi.org/10.1093/sysbio/syy032 PMID: 29718447

71. Stamatakis A. RAxML-VI-HPC: maximum likelihood-based phylogenetic analyses with thousands of taxa and mixed models. Bioinformatics. 2006; 22: 2688–2690. https://doi.org/10.1093/bioinformatics/btl446 PMID: 16928733

72. Silvestro D, Michalak I. raxmlGUI: a graphical front-end for RAxML. Org Divers Evol. 2012; 12: 335–337. https://doi.org/10.1007/s13127-011-0056-0

73. Cracraft J, Barker FK, Braun M, Harshman J, Dyke GJ, Feinstein J, et al. Phylogenetic relationships among modern birds (Neornithes). Toward an avian tree of life. In: Cracraft J, Donoghue MJ, editors. Assembling the tree of life. Oxford: Oxford University Press; 2004. pp. 468–489.

74. Manegold A. Earliest fossil record of the Certhioidea (treecreepers and allies) from the early Miocene of Germany. J Ornithol. 2008; 149: 223–228. https://doi.org/10.1007/s10336-007-0263-9

75. Volkova NV, Zelenkov NV. A scansorial passerine bird (Passeriformes, Certhioidea) from the uppermost Lower Miocene of Eastern Siberia. Paleontol J. 2018; 52: 58–65. https://doi.org/10.1134/S0031030118010148

76. Agustí J, Cabrera L, Garcés M, Krijgsman W, Oms O, Parés JM. A calibrated mammal scale for the Neogene of Western Europe. State of the art. Earth-Sci Rev. 2001; 52: 247–260. https://doi.org/10.1016/S0012-8252(00)00025-8
77. Benton MJ, Donoghue PC, Asher RJ. Calibrating and constraining molecular clocks. In: Hedges SB, Kumar S, editors. The timetree of life. Oxford: Oxford University Press; 2009. pp. 35–86.
78. Claramunt S, Cracraft J. A new time tree reveals Earth history's imprint on the evolution of modern birds. Sci Adv. 2015; 1: e1501005. https://doi.org/10.1126/sciadv.1501005 PMID: 26824065
79. Barker FK. Monophyly and relationships of wrens (Aves: Troglodytidae): a congruence analysis of heterogeneous mitochondrial and nuclear DNA sequence data. Mol Pylogegenetics Evol. 2004; 31: 486–504. https://doi.org/10.1016/j.ympev.2003.08.005 PMID: 15062790
80. Zhao M, Alström P, Olsson U, Qu Y, Lei F. Phylogenetic position of the Wallcreeper *Tichodroma muraria*. J Ornithol. 2016; 157: 913–918. https://doi.org/10.1007/s10336-016-1340-8
81. Walker JD, Geissman JW, Bowring SA, Babcock LE (compilers). Geologic Time Scale v.5.0. Geological Society of America. 2018. https://doi.org/10.1130/2018.CTS005R3C: Cited 20 December 2018.
82. Rambaut A. FigTree. Tree Figure Drawing Tool, version 1.4.3. 2016. Available from: http://tree.bio.ed.ac.uk/software/figtree. Cited 10 August 2017.
83. Weir J, Schluter D. Calibrating the avian molecular clock. Mol Ecol. 2008; 17: 2321–2328. https://doi.org/10.1111/j.1365-294X.2008.03742.x PMID: 18422932
84. Barker FK, Benesh MK, Vandergon AJ, Lanyon SM. Contrasting Evolutionary Dynamics and Information Content of the Avian Mitochondrial Control Region and ND2 Gene. PLOS ONE. 2012; 7: e46403. https://doi.org/10.1371/journal.pone.0046403 PMID: 23071562
85. Päckert M, Martens J, Tietze DT, Dietzen C, Wink M, Kvist L. Calibration of a molecular clock in tits (Paridae)—Do nucleotide substitution rates of mitochondrial genes deviate from the 2% rule? Mol Phylogenetics Evol. 2007; 44, 1–14. https://doi.org/10.1016/j.ympev.2007.03.006 PMID: 17512759
86. Pacheco MA, Battistuzzi FU, Lentino M, Aguilar RF, Kumar S, Escalante AA. Evolution of modern birds revealed by mitogenomics: timing the radiation and origin of major orders. Mol Biol Evol. 2011; 28: 1927–1942. https://doi.org/10.1093/molbev/msr014 PMID: 21242529
87. Lavinia PD, Kerr KCK, Tubaro PL, Hebert PDN, Lijtmaer DA. Calibrating the molecular clock beyond cytochrome *b*: assessing the evolutionary rate of COI in birds. J Avian Biol. 2016; 47: 84–91. https://doi.org/10.1111/jav.00766
88. Bininda-Emonds ORP. Fast genes and slow clades: comparative rates of molecular evolution in mammals. Evol Bioinform. 2007; 3: 59–85. https://doi.org/10.1177/117693430700300008
89. Eo SH, DeWoody JA. Evolutionary rates of mitochondrial genomes correspond to diversification rates and to contemporary species richness in birds and reptiles. Proc R Soc Lond B, Biol Sci. 2010; 277: 3587–3592. https://doi.org/10.1098/rspb.2010.0965 PMID: 20610427
90. Pereira SL, Baker AJ. A Mitogenomic Timescale for Birds Detects Variable Phylogenetic Rates of Molecular Evolution and Refutes the Standard Molecular Clock. Mol Biol Evol. 2006; 23: 1731–1740. https://doi.org/10.1093/molbev/msl038 PMID: 16774978
91. Nabholz B, Glémin S, Galtier N. The erratic mitochondrial clock: variations of mutation rate, not population size, affect mtDNA diversity across birds and mammals. BMC Evol Biol. 2009; 9: 54. https://doi.org/10.1186/1471-2148-9-54 PMID: 19284537
92. Quillfeldt P. Body mass is less important than bird order in determining the molecular rate for mitochondrial DNA. Mol Ecol. 2017; 26: 2426–2429. https://doi.org/10.1111/mec.14103 PMID: 28391643
93. Smith BT, Klicka J. Examining the role of effective population size on mitochondrial and multilocus divergence time discordance in a song bird. PLoS One. 2010; 8: e55161. https://doi.org/10.1371/journal.pone.0055161 PMID: 23457463
94. Lerner HRL, Meyer M, James HF, Hofreiter M, Fleischer RC. Multilocus resolution of phylogeny and timescale in the extant adaptive radiation of Hawaiian honeycreepers. Curr Biol. 2011; 21: 1–7. https://doi.org/10.1016/j.cub.2010.11.056
95. Joseph L, Toon A, Nyári AS, Longmore NW, Rowe KMC, Haryoko T, et al. A new synthesis of the molecular systematics and biogeography of honeyeaters (Passeriformes: Meliphagidae) highlights biogeographical and ecological complexity of a spectacular avian radiation. Zool Scr. 2014; 43: 235–248. https://doi.org/10.1111/zsc.12049
96. Smith BT, Klicka J. Examining the Role of Effective Population Size on Mitochondrial and Multilocus Divergence Time Discordance in a Songbird. PLoS One, 2013; 8: e55161. https://doi.org/10.1371/journal.pone.0055161 PMID: 23457463
97. Slowinski JB, Arbogast BS. Is the rate of molecular evolution inversely related to body size? Syst Biol. 1999; 48: 396–399. https://doi.org/10.1080/106351599260364 PMID: 12066715

98. Thomson CE, Gilbert JDJ, Brooke MdL. Cytochrome b divergence between avian sister species is linked to generation length and body mass. PLoS One. 2014; 9: e85006. https://doi.org/10.1371/journal.pone.0085006 PMID: 24505250

99. Nabholz B, Lanfear R, Fuchs J. Body mass-corrected molecular rate for bird mitochondrial DNA. Mol Ecol. 2016; 25: 4438–4449. https://doi.org/10.1111/mec.13780 PMID: 27483387

100. Mayr G, Manegold A. The oldest European fossil songbird from the early Oligocene of Germany. Naturwissenschaften. 2004; 91: 173–177. https://doi.org/10.1007/s00114-004-0509-9 PMID: 15085274

101. Mayr G. The age of the crown group of passerine birds and its evolutionary significance–molecular calibrations versus the fossil record. System Biodivers. 2013; 11: 7–13. https://doi.org/10.1080/14772000.2013.765521

102. Ho SYW, Larson G. Molecular clocks: when times are a-changin'. Trends Genet. 2006; 22: 79–83. https://doi.org/10.1016/j.tig.2005.11.006 PMID: 16356585

103. Ho SYW, Saarma U, Barnett R, Haile J, Shapiro B. The effect of inappropriate calibration: three case studies in molecular ecology. PLoS One. 2008; 3: e1615. https://doi.org/10.1371/journal.pone.0001615 PMID: 18286172

104. Hijmans RJ, Cameron S, Parra J, Jones P, Jarvis A, Richardson K [cited 14 October 2019]. WorldClim—Global Climate Data. Free climate data for ecological modeling and GIS. Version 1.4. Available from: http://worldclim.org/version1.

105. GBIF.org [cited 21 June 2017]. GBIF occurrence download for preserved specimens of *Troglodytes troglodytes* (Linnaeus, 1758). Available from: https://www.gbif.org/occurrence/download/0103016-160910150852091. https://doi.org/10.15468/dl.bztotw

106. National Science Foundation [cited 21 June 2017]. VertNet occurrence download for mappable specimens of *Troglodytes troglodytes*. Available from: http://vertnet.org/.

107. Mayr E, Greenway JC jr., editors. Check-List of Birds of the World. A continuation of the work of James L. Peters. Vol. IX. Cambridge, Massachusetts: Museum of Comparative Zoology; 1960.

108. Brown JL. SDM toolbox: a python-based GIS toolkit for landscape genetic, biogeographic and species distribution model analyses. Methods Ecol Evol. 2014; 5: 694–700. https://doi.org/10.1111/2041-210X.12200

109. Braconnot P, Harrison SP, Kageyama M, Bartlein PJ, Masson-Delmotte V, Abe-Ouchi A, et al. Evaluation of climate models using palaeoclimatic data. Nat Clim Change. 2012; 2: 417–424. https://doi.org/10.1038/nclimate1456

110. Otto-Bliesner BL, Brady EC, Clauzet G, Tomas R, Levis S, Kothavala Z. Last glacial maximum and Holocene climate in CCSM3. J Clim. 2006; 19: 2526–2544. https://doi.org/10.1175/JCLI3748.1

111. Hasumi H, Emori S, editors. K-1 coupled GCM (MIROC) description. K-1 Technical Report No. 1. Tokyo: Center for Climate System Research, University of Tokyo; 2004.

112. Phillips SJ, Anderson RP, Schapire RE. Maximum entropy modeling of species geographic distributions. Ecol Modelling; 2006; 190: 231–259. https://doi.org/10.1016/j.ecolmodel.2005.03.026

113. Phillips SJ, Dudík M. Modeling of species distributions with Maxent: new extensions and a comprehensive evaluation. Ecography. 2008; 31: 161–175. https://doi.org/10.1111/j.0906-7590.2008.5203.x

114. Phillips SJ, Dudík M, Schapire RE. Maxent software for modeling species niches and distributions (Version 3.4.1). Available from: http://biodiversityinformatics.amnh.org/open_source/maxent/. Cited 01 January 2019.

115. Ling CX, Huang J, Zhang H. AUC: a better measure than accuracy in comparing learning algorithms. In: Xiang Y, Chaib-draa B, editors. Advances in Artificial Intelligence. Canadian AI 2003. Lecture Notes in Computer Science (Lecture Notes in Artificial Intelligence), vol 2671. Berlin, Heidelberg: Springer; 2003. pp. 329–341. https://doi.org/10.1007/3-540-44886-1_25

116. Elith J, Graham CH, Anderson RP, Dudík M, Ferrier S, Guisan A, et al. Novel methods improve prediction of species' distributions from occurrence data. Ecography. 2006; 29: 129–151. https://doi.org/10.1111/j.2006.0906–7590.04596.x

117. Päckert M, Martens J, Nazarenko AA, Kosuch J, Veith M. Phylogenetic signal in song of crests and kinglets (Aves: *Regulus*). Evol. 2003; 57: 616–629. https://doi.org/10.1111/j.0014-3820.2003.tb01553.x

118. Pasquet E, Barker FK, Martens J, Tillier A, Cruaud C, Cibois A. Evolution within the nuthatches (Sittidae: Aves, Passeriformes): molecular phylogeny, biogeography, and ecological perspectives. J Ornithol. 2014; 155: 755–765. https://doi.org/10.1007/s10336-014-1063-7

119. Smith BT, Klicka J. The profound influence of the Late Pliocene Panamanian uplift on the exchange, diversification, and distribution of New World birds. Ecography. 2010; 33: 333–342. https://doi.org/10.1111/j.1600-0587.2009.06335.x

120. Martens J, Tietze DT, Päckert M. Phylogeny, biodiversity, and species limits of passerine birds in the Sino-Himalayan region—A critical review. Ornithol Monogr. 2011; 70: 64–94. https://doi.org/10.1525/om.2011.70.1.64

121. Päckert M, Martens J, Sun Y-H, Severinghaus LL, Nazarenko AA, Ting J, et al. Horizontal and elevational phylogeographic patterns of Himalayan and Southeast Asian forest passerines (Aves: Passeriformes). J Biogeogr. 2012; 39: 556–573. https://doi.org/10.1111/j.1365-2699.2011.02606.x

122. Päckert M, Martens J, Sun Y-H. Cradles of evolution–Hugo Weigold's biogeography of Tibet and the Sino-Himalayas in the light of molecular systematics and phylogeography. In: Hartmann M, Weipert J, editors. Biodiversity and Natural Heritage of the Himalaya. Vol. VI. Erfurt: Verein der Freunde und Förderer des Naturkundemuseums Erfurt; 2018. pp. 153–162.

123. Päckert M, Martens J, Sun Y-H, Tietze DT. Evolutionary history of passerine birds (Aves: Passeriformes) from the Qinghai-Tibetan plateau: from a pre-Quarternary perspective to an integrative biodiversity assessment. J Ornithol. 2015; 156: 355–365. https://doi.org/10.1007/s10336-015-1185-6

124. Brito PH. The influence of Pleistocene glacial refugia on tawny owl genetic diversity and phylogeography in western Europe. Mol Ecol. 2005; 14: 3077–3094. https://doi.org/10.1111/j.1365-294X.2005.02663.x PMID: 16101775

125. Brito PH. Contrasting patterns of mitochondrial and microsatellite genetic structure among Western European populations of tawny owls (*Strix aluco*). Mol Ecol. 2007; 16: 3423–3437. https://doi.org/10.1111/j.1365-294X.2007.03401.x PMID: 17688543

126. Doña J, Ruiz-Ruano FJ, Jovani R. DNA barcoding of Iberian Peninsula and North Africa Tawny Owls *Strix aluco* suggests the Strait of Gibraltar as an important barrier for phylogeography. Mitochondrial DNA Part A. 2016; 27: 4475–4478. https://doi.org/10.3109/19401736.2015.1089573 PMID: 26465068

127. Pons JM, Olioso G, Cruaud C, Fuchs J. Phylogeography of the Eurasian green woodpecker (*Picus viridis*). J Biogeogr. 2011; 38: 311–325. https://doi.org/10.1111/j.1365-2699.2010.02401.x

128. Song G, Zhang R, Alström P, Irestedt M, Cai T, Qu Y, et al. Complete taxon sampling of the avian genus *Pica* (magpies) reveals ancient relictual populations and synchronous Late-Pleistocene demographic expansion across the Northern Hemisphere. J Avian Biol. 2018; 49: e01612. https://doi.org/10.1111/jav.01612

129. Pentzold S, Tritsch C, Martens J, Tietze DT, Giacalone G, Valvo ML, et al. Where is the line? Phylogeography and secondary contact of western Palearctic coal tits (*Periparus ater*: Aves, Passeriformes, Paridae). Zool Anz. 2013; 252: 367–382. https://doi.org/10.1016/j.jcz.2012.10.003

130. Procházka P, Stokke BG, Jensen H, Fainová D, Bellinvia E, Fossøy F, et al. Low genetic differentiation among reed warbler *Acrocephalus scirpaceus* populations across Europe. J Avian Biol. 2011; 42: 103–113. https://doi.org/10.1111/j.1600-048X.2010.05161.x

131. Olsson U, Rguibi-Idrissi H, Copete JL, Matos JLA, Provost P, Amezian M, et al. Mitochondrial phylogeny of the Eurasian/African reed warbler complex (*Acrocephalus*, Aves). Disagreement between morphological and molecular evidence and cryptic divergence: A case for resurrecting *Calamoherpe ambigua* Brehm 1857. Mol Phylogenetics Evol. 2016; 102: 30–44. https://doi.org/10.1016/j.ympev.2016.05.026 PMID: 27233439

132. Tietze DT, Martens J, Sun YH. Molecular phylogeny of treecreepers (*Certhia*) detects hidden diversity. Ibis. 2006; 148: 477–488. https://doi.org/10.1111/j.1474-919X.2006.00547.x

133. Hourlay F, Libois R, D'Amico F, Sarà M, O'Halloran J, Michaux JR. Evidence of a highly complex phylogeographic structure on a specialist river bird species, the dipper (*Cinclus cinclus*). Mol Phylogenetics Evol. 2008; 49: 435–444. https://doi.org/10.1016/j.ympev.2008.07.025 PMID: 18762260

134. Pons JM, Thibault JC, Aymí R, Grussu M, Muntaner J, Olioso G, et al. The role of western Mediterranean islands in the evolutionary diversification of the spotted flycatcher *Muscicapa striata*, a long-distance migratory passerine species. J Avian Biol. 2016; 47:, 386–398. https://doi.org/10.1111/jav.00859

135. Hogner S, Sæther SA, Borge T, Bruvik T, Johnsen A, Sætre GP. Increased divergence but reduced variation on the Z chromosome relative to autosomes in *Ficedula* flycatchers: differential introgression or the faster-Z effect?. Ecol Evol. 2012; 2: 379–396. https://doi.org/10.1002/ece3.92 PMID: 22423331

136. Moyle RG, Hosner PA, Jones AW, Outlaw DC. Phylogeny and biogeography of *Ficedula* flycatchers (Aves: Muscicapidae): novel results from fresh source material. Mol Phylogenetics Evol. 2015; 82: 87–94. https://doi.org/10.1016/j.ympev.2014.09.029 PMID: 25307119

137. Marshall HD, Baker AJ. Colonization history of Atlantic island common chaffinches (*Fringilla coelebs*) revealed by mitochondrial DNA. Mol Phylogenetics Evol. 1999; 11: 201–212. https://doi.org/10.1006/mpev.1998.0552 PMID: 10191065

138. Griswold C.K., Baker A.J., 2002. Time to the most recent common ancestor and divergence times of populations of common chaffinches (Fringilla coelebs) in Europe and North Africa: insights into

Pleistocene refugia and current levels of migration. Evol. 56, 143–153. https://doi.org/10.1111/j.0014-3820.2002.tb00856.x.

139. Parchman TL, Edelaar P, Uckele K, Mezquida ET, Alonso D, Jahner JP, et al. Resource stability and geographic isolation are associated with genome divergence in western Palearctic crossbills. J Evol Biol. 2018; 31: 1715–1731. https://doi.org/10.1111/jeb.13367 PMID: 30125437

140. Zangari F, Cimmaruta R, Nascetti G. Genetic relationships of the western Mediterranean painted frogs based on allozymes and mitochondrial markers: evolutionary and taxonomic inferences (Amphibia, Anura, Discoglossidae). Biol J Linn Soc. 2006; 87: 515–536. https://doi.org/10.1111/j.1095-8312.2006.00585.x

141. Calvignac S, Hughes S, Tougard C, Michaux J, Thevenot M, Philippe M, et al. Ancient DNA evidence for the loss of a highly divergent brown bear clade during historical times. Mol Ecol. 2008; 17:, 1962–1970. https://doi.org/10.1111/j.1365-294X.2008.03631.x PMID: 18363668

142. Calvignac S, Hughes S, Hänni C. Genetic diversity of endangered brown bear (*Ursus arctos*) populations at the crossroads of Europe, Asia and Africa. Divers Distrib. 2009; 15: 742–750. https://doi.org/10.1111/j.1472-4642.2009.00586.x

143. Leite JV, Álvares F, Velo-Antón G, Brito JC, Godinho R. (2015). Differentiation of North African foxes and population genetic dynamics in the desert—insights into the evolutionary history of two sister taxa, *Vulpes rueppellii* and *Vulpes vulpes*. Org Divers Evol. 2015; 15: 731–745. https://doi.org/10.1007/s13127-015-0232-8

144. Mattucci F, Oliveira R, Lyons LA, Alves PC, Randi E. European wildcat populations are subdivided into five main biogeographic groups: consequences of Pleistocene climate changes or recent anthropogenic fragmentation? Ecol Evol. 2016; 6: 3–22. https://doi.org/10.1002/ece3.1815 PMID: 26811770

145. Escudero M, Vargas P, Valcárcel V, Luceño M. Strait of Gibraltar: an effective gene-flow barrier for wind-pollinated *Carex helodes* (Cyperaceae) as revealed by DNA sequences, AFLP, and cytogenetic variation. Am J Bot. 2008; 95: 745–755. https://doi.org/10.3732/ajb.2007342 PMID: 21632400

146. Gantenbein B, Largiadèr CR. The phylogeographic importance of the Strait of Gibraltar as a gene flow barrier in terrestrial arthropods: a case study with the scorpion *Buthus occitanus* as model organism. Mol Phylogenetics Evol. 2003; 28: 119–130. https://doi.org/10.1016/S1055-7903(03)00031-9

147. Jaramillo-Correa JP, Grivet D, Terrab A, Kurt Y, De-Lucas AI, Wahid N, et al. The Strait of Gibraltar as a major biogeographic barrier in Mediterranean conifers: a comparative phylogeographic survey. Mol Ecol. 2010; 19: 5452–5468. https://doi.org/10.1111/j.1365-294X.2010.04912.x PMID: 21073588

148. García-Alix A, Minwer-Barakat R, Martín Suárez E, Freudenthal M, Aguirre J, Kaya F. Updating the Europe–Africa small mammal exchange during the late Messinian. J Biogeogr. 2016; 43: 1336–1348. https://doi.org/10.1111/jbi.12732

149. Gibert L, Scott GR, Montoya P, Ruiz-Sánchez FJ, Morales J, Luque L, et al. Evidence for an African-Iberian mammal dispersal during the pre-evaporitic Messinian. Geology. 2013; 41: 691–694. https://doi.org/10.1130/G34164.1

150. Carranza S, Arnold EN, Pleguezuelos JM. Phylogeny, biogeography, and evolution of two Mediterranean snakes, *Malpolon monspessulanus* and *Hemorrhois hippocrepis* (Squamata, Colubridae), using mtDNA sequences. Mol Phylogenetics Evol. 2006; 40: 532–546. https://doi.org/10.1016/j.ympev.2006.03.028 PMID: 16679033

151. Mendes J, Harris DJ, Carranza S, Salvi D. Biogeographical crossroad across the Pillars of Hercules: Evolutionary history of *Psammodromus* lizards in space and time. J Biogeogr. 2017; 44: 2877–2890. https://doi.org/10.1111/jbi.13100

152. Recuero E, Iraola A, Rubio X, Machordom A, García-París M. Mitochondrial differentiation and biogeography of *Hyla meridionalis* (Anura: Hylidae): an unusual phylogeographical pattern. J Biogeogr. 2007; 34: 1207–1219. https://doi.org/10.1111/j.1365-2699.2007.01688.x

153. Hewitt GM. Quaternary phylogeography: the roots of hybrid zones. Genetica. 2011; 139: 617–638. https://doi.org/10.1007/s10709-011-9547-3 PMID: 21234647

154. Graciá E, Vargas-Ramírez M, Delfino M, Anadón JD, Giménez A, Fahd S, et al. Expansion after expansion: dissecting the phylogeography of the widely distributed spur-thighed tortoise, Testudo graeca (Testudines: Testudinidae). Biol J Linn Soc. 2017; 121: 641–654. https://doi.org/10.1093/biolinnean/blx007

155. Tamar K, Carranza S, Sindaco R, Moravec J, Trape JF, Meiri S. Out of Africa: Phylogeny and biogeography of the widespread genus *Acanthodactylus* (Reptilia: Lacertidae). Mol Phylogenetics Evol. 2016; 103: 6–18. https://doi.org/10.1016/j.ympev.2016.07.003 PMID: 27395778

156. Guillaumet A, Crochet PA, Pons JM. Climate-driven diversification in two widespread *Galerida* larks. BMC Evol Biol. 2008; 8: 32. https://doi.org/10.1186/1471-2148-8-32 PMID: 18230151

157. Ben Faleh AR, Granjon L, Tatard C, Othmen AB, Said K, Cosson JF. Phylogeography of the Greater Egyptian Jerboa (*Jaculus orientalis*) (Rodentia: Dipodidae) in Mediterranean North Africa. J Zool. 2012; 286: 208–220. https://doi.org/10.1111/j.1469-7998.2011.00868.x

158. Cosson JF, Hutterer R, Libois R, Sara M, Taberlet P, Vogel P. (2005). Phylogeographical footprints of the Strait of Gibraltar and Quaternary climatic fluctuations in the western Mediterranean: a case study with the greater white-toothed shrew, *Crocidura russula* (Mammalia: Soricidae). Mol Ecol. 2005; 14: 1151–1162. https://doi.org/10.1111/j.1365-294X.2005.02476.x PMID: 15773942

159. deMenocal PB. Plio-Pleistocene African Climate. Science. 1995; 270: 53–59. https://doi.org/10.1126/science.270.5233.53 PMID: 7569951

160. deMenocal PB. African climate change and faunal evolution during the Pliocene–Pleistocene. Earth Planet Sci Lett. 2004; 220: 3–24. https://doi.org/10.1016/S0012-821X(04)00003-2

161. Bertoldi R, Rio D, Thunell R. Pliocene-Pleistocene vegetational and climatic evolution of the south-central Mediterranean. Palaeogeogr Palaeoclimatol Palaeoecol. 1989; 72: 263–275. https://doi.org/10.1016/0031-0182(89)90146-6

162. Prendergast AL, Stevens RE, O'Connell TC, Fadlalak A, Touati M, Al-Mzeine A, et al. Changing patterns of eastern Mediterranean shellfish exploitation in the Late Glacial and Early Holocene: Oxygen isotope evidence from gastropod in Epipaleolithic to Neolithic human occupation layers at the Haua Fteah cave, Libya. Quat Int. 2016; 407: 80–93. https://doi.org/10.1016/j.quaint.2015.09.035

163. Prendergast AL, Stevens RE, O'Connell TC, Hill EA, Hunt CO, Barker GW. A late Pleistocene refugium in Mediterranean North Africa? Palaeoenvironmental reconstruction from stable isotope analyses of land snail shells (Haua Fteah, Libya). Quat Sci Rev. 2016; 139: 94–109. https://doi.org/10.1016/j.quascirev.2016.02.014

164. Reade H, O'Connell TC, Barker G, Stevens RE. Pleistocene and Holocene palaeoclimates in the Gebel Akhdar (Libya) estimated using herbivore tooth enamel oxygen isotope compositions. Quat Int. 2016; 404: 150–162. https://doi.org/10.1016/j.quaint.2015.10.009

165. Tritsch C, Stuckas H, Martens J, Pentzold S, Kvist L, Lo Valvo M, et al. Gene flow in the European coal tit, *Periparus ater* (Aves: Passeriformes): low among Mediterranean populations but high in a continental contact zone. Biol J Linn Soc. 2018; 124: 319–338. https://doi.org/10.1093/biolinnean/bly043

166. Haring E, Gamauf A, Kryukov A. Phylogeographic patterns in widespread corvid birds. Mol Phylogenetics Evol. 2007; 45: 840–862. https://doi.org/10.1016/j.ympev.2007.06.016 PMID: 17920300

167. Irwin DE, Rubtsov AS, Panov EN. Mitochondrial introgression and replacement between yellowhammers (*Emberiza citrinella*) and pine buntings (*Emberiza leucocephalos*)(Aves: Passeriformes). Biol J Linn Soc. 2009; 98: 422–438. https://doi.org/10.1111/j.1095-8312.2009.01282.x

168. Albrecht F, Hering J, Fuchs E, Martens J, Päckert M. Entfernte Verwandtschaft in der Cyrenaika–die phylogenetische Stellung des Eurasischen Zaunkönigs *Troglodytes troglodytes juniperi* aus Libyen. Vogelwarte. 2017; 55: 360–361.

169. Batista V, Harris DJ. Genetic variation in *Pleurodeles waltl* Michahelles, 1830 across the Strait of Gibraltar derived from mitochondrial DNA sequences. Herpetozoa. 2004; 16: 166–168.

170. Garcia-Mudarra JL, Ibanez C, Juste J. The Straits of Gibraltar: barrier or bridge to Ibero-Moroccan bat diversity? Biol J Linn Soc. 2009; 96: 434–450. https://doi.org/10.1111/j.1095-8312.2008.01128.x

171. Lauga B, Cagnon C, D'Amico F, Karama S, Mouchès C. Phylogeography of the white-throated dipper *Cinclus cinclus* in Europe. J Ornithol. 2005; 146: 257–262. https://doi.org/10.1007/s10336-005-0087-4

172. Hernández MÁ, Campos F, Santamaría T, Corrales L, Rojo MA, Dias S. Genetic differences among Iberian White-throated dipper *Cinclus cinclus* populations based on the cytochrome b sequence. Ardeola. 2012; 59: 111–123. https://doi.org/10.13157/arla.59.1.2012.111

173. Neto JM, Arroyo JL, Bargain B, Monrós JS, Mátrai N, Procházka P, et al. Phylogeography of a habitat specialist with high dispersal capability: The Savi's Warbler *Locustella luscinioides*. PLOS ONE. 2012; 7:e38497. https://doi.org/10.1371/journal.pone.0038497 PMID: 22701653

174. Drovetski SV, Fadeev IV, Raković M, Lopes RJ, Boano G, Pavia M, et al. A test of the European Pleistocene refugial paradigm, using a Western Palaearctic endemic bird species. Proc R Soc Lond B, Biol Sci. 2018; 285: 20181606. https://doi.org/10.1098/rspb.2018.1606 PMID: 30355709

175. Purroy FJ, Purroy-Balda J. Chochín, *Troglodytes troglodytes*. In: Martí R, Del Moral JC, editors. Atlas de las Aves Reproductoras de España. Madrid: Dirección General de Conservación de la Naturaleza, Sociedad Española de Ornitología; 2003. pp. 408–409. Available from: https://www.miteco.gob.es/es/biodiversidad/temas/inventarios-nacionales/chochin_tcm30-100188.pdf. Cited 20 February 2019.

176. Barros F, Matias R. Carriça, *Troglodytes troglodytes*. In: Equipa Atlas, editor. Atlas das Aves Nidificantes em Portugal (1999–2005). Lisbon: Instituto da Conservação da Natureza e da Biodiversidade, Sociedade Portuguesa para o Estudo das Aves, Parque Natural de Madeira e Secretaria Regional do Ambiente e do Mar. Assírio & Alvim; 2008. pp. 352–353.
